# The transcription factor PRO44 and the histone chaperone ASF1 regulate distinct aspects of multicellular development in the filamentous fungus *Sordaria macrospora*

**DOI:** 10.1186/s12863-018-0702-z

**Published:** 2018-12-13

**Authors:** David Immanuel Schumacher, Ramona Lütkenhaus, Florian Altegoer, Ines Teichert, Ulrich Kück, Minou Nowrousian

**Affiliations:** 1 Lehrstuhl für Allgemeine und Molekulare Botanik, Ruhr-Universität Bochum, 44780 Bochum, Germany; 2 LOEWE-Zentrum für Synthetische Mikrobiologie & Department of Chemistry, Philipps University of Marburg, Marburg, Germany

**Keywords:** *Sordaria macrospora*, Ascomycetes, Fruiting body development, Sexual development, Histone chaperone, Transcription factor, RNA-seq, Laser microdissection

## Abstract

**Background:**

Fungal fruiting bodies are complex three-dimensional structures that are formed to protect and disperse the sexual spores. Their morphogenesis requires the concerted action of numerous genes; however, at the molecular level, the spatio-temporal sequence of events leading to the mature fruiting body is largely unknown. In previous studies, the transcription factor gene *pro44* and the histone chaperone gene *asf1* were shown to be essential for fruiting body formation in the ascomycete *Sordaria macrospora*. Both PRO44 and ASF1 are predicted to act on the regulation of gene expression in the nucleus, and mutants in both genes are blocked at the same stage of development. Thus, we hypothesized that PRO44 and ASF1 might be involved in similar aspects of transcriptional regulation. In this study, we characterized their roles in fruiting body development in more detail.

**Results:**

The PRO44 protein forms homodimers, localizes to the nucleus, and is strongly expressed in the outer layers of the developing young fruiting body. Analysis of single and double mutants of *asf1* and three other chromatin modifier genes, *cac2*, *crc1*, and *rtt106*, showed that only *asf1* is essential for fruiting body formation whereas *cac2* and *rtt106* might have redundant functions in this process. RNA-seq analysis revealed distinct roles for *asf1* and *pro44* in sexual development, with *asf1* acting as a suppressor of weakly expressed genes during morphogenesis. This is most likely not due to global mislocalization of nucleosomes as micrococcal nuclease-sequencing did not reveal differences in nucleosome spacing and positioning around transcriptional start sites between Δasf1 and the wild type. However, bisulfite sequencing revealed a decrease in DNA methylation in Δasf1, which might be a reason for the observed changes in gene expression. Transcriptome analysis of gene expression in young fruiting bodies showed that *pro44* is required for correct expression of genes involved in extracellular metabolism. Deletion of the putative transcription factor gene *asm2*, which is downregulated in young fruiting bodies of Δpro44, results in defects during ascospore maturation.

**Conclusions:**

In summary, the results indicate distinct roles for the transcription factor PRO44 and the histone chaperone ASF1 in the regulation of sexual development in fungi.

**Electronic supplementary material:**

The online version of this article (10.1186/s12863-018-0702-z) contains supplementary material, which is available to authorized users.

## Background

Fungi are among several groups of eukaryotes that are able to develop complex multicellular structures [1–4]. One example for such structures are the fruiting bodies of filamentous ascomycetes (Pezizomycotina) [5–7]. Within fruiting bodies, sexual spores are formed, and the surrounding fruiting body structures can protect the spores and facilitate their distribution. Ascomycete fruiting bodies contain a number of cell types that are not found in vegetative mycelium [8–10]. The morphology of fruiting body development was studied in detail in the model organisms *Neurospora crassa* and *Sordaria macrospora* [8, 10]. In the homothallic (self-fertile) *S. macrospora*, fruiting body development is possible for a single strain without the need of a compatible mating partner. Sexual development starts with the formation of female sexual structures, the ascogonial coils, which are enveloped by sterile hyphae forming a young fruiting body called protoperithecium. The sterile, outer layers of the protoperithecium differentiate into a darkly pigmented outer layer and a less pigmented inner wall region, with additional cell types differentiating during the transition to a mature fruiting body (perithecium) [10]. The differentiation of fruiting bodies in fungi is accompanied by massive transcriptional changes that are thought to enable the differentiation of specific cell types [6, 11]. At the molecular level, the regulatory principles that mediate correct spatio-temporal gene expression during fruiting body development are not clear yet. However, in recent years, a lot of progress has been made in identifying developmental genes that encode factors potentially influencing transcription; and this includes specific transcription factors as well as chromatin modifiers.

Well-studied transcription factor genes that are required for fruiting body development in a number of ascomycete species include, for example, several mating type genes [12, 13], genes encoding proteins of the velvet family [14], and homologs of the zinc cluster transcription factor PRO1 [15–20]. Another example of a conserved ascomycete transcription factor with a role in development is the GATA factor NsdD/SUB-1/PRO44 (from now on called PRO44 if not referring to a differently named specific ortholog). The corresponding gene was independently identified as affected in developmental mutants of *Aspergillus nidulans* (*nsdD*), *Neurospora crassa* (*sub-1*), and *Sordaria macrospora* (*pro44*) [17, 20, 21]. Furthermore, orthologs were shown to be essential for sexual development in *Aspergillus fumigatus*, *Botrytis cinerea*, and *Trichoderma reesei* [22–24]. These data indicate a conserved role for PRO44 in ascomycete fruiting body formation, as it is required for this process in evolutionary diverse species from the Sordariomycetes (*N. crassa, S. macrospora*, and *T. reesei*), Eurotiomycetes (*A. nidulans* and *A. fumigatus*), and Leotiomycetes (*B. cinerea*). In addition, the *pro44* homolog from *Pyronema confluens*, a member of the early-diverging Pezizomycete lineage, can complement the developmental defects of an *S. macrospora* Δpro44 mutant, suggesting a conserved molecular function that might have been already present in the last common ancestor of filamentous ascomycetes [25]. In addition to its role in sexual development, *pro44* orthologs were shown to be involved in the regulation of light-dependent processes in *N. crassa* and *B. cinerea* [22, 26, 27]. For both species, transcriptome analyses identified genes that are differentially regulated in the absence of the transcription factor gene, and for *N. crassa*, ChIP (chromatin immunoprecipitation)-seq analyses identified direct genomic targets bound by the PRO44 ortholog SUB-1 [22, 26, 27]. However, these studies were performed with mycelia not grown under conditions for sexual development. Therefore the influence of *pro44* orthologs on gene expression during fruiting body formation remains unknown. Previous studies in *S. macrospora* have shown that *pro44* itself is strongly expressed in young fruiting bodies at the level of transcription, and that this expression is dependent on the transcription factor gene *pro1* [28]. Therefore, one aim of this study was to determine the transcriptome of the *S. macrospora* Δpro44 mutant in developing fruiting bodies, and to compare this with transcriptomes of other developmental mutants and the wild type at the same developmental stages to characterize the role of *pro44* in potential regulatory networks. Specifically, we aimed to address the question whether there might be an overlap between the function of transcription factors like PRO44 and chromatin modifiers in fruiting body development.

For chromatin modifiers, little is known with respect to their role in fruiting body development. In general, chromatin modifiers can mediate changes of the DNA itself, e.g. through methylation, changes in nucleosome positioning, incorporation of histone variants, or numerous histone modifications [29–32]. In recent years, several genes with potential functions in chromatin modification were found to be involved in fruiting body morphogenesis, pointing to an important role for regulatory events at chromatin level in this process [33–36]. One such factor is the histone chaperone ASF1 that was identified as a developmental gene in *S. macrospora* [37]. Histone chaperones are a heterogeneous group of proteins that can be characterized by their ability to bind non-nucleosomal histones in vivo, and mediate the assembly of nucleosomes on DNA in vitro [38]. They are involved in all chromatin-dependent processes, e.g. DNA replication, repair, and transcription [39]. ASF1 is a conserved histone chaperone specific for histones H3 and H4 [40]. Interestingly, deletion of *asf1* is lethal in many organisms where it was investigated, and so far, *S. macrospora* and the plant *Arabidopsis thaliana* are the only multicellular organisms where Δasf1 mutants are viable [37, 41]. However, the *S. macrospora* mutant is unable to develop mature fruiting bodies (perithecia). It only forms young, immature fruiting bodies (protoperithecia) that do not contain sexual spores [37], similar to the pro44 mutant, which is also blocked a the stage of protoperithecium formation [21]. Several developmental genes are transcriptionally deregulated in the Δasf1 mutant strain, but the extent to which ASF1 might regulate gene expression during fungal development and the molecular mechanisms behind this are not yet clear. Therefore, one aim of our analyses was to identify transcriptional changes in the *S. macrospora* Δasf1 mutant on a genome-wide basis. Another aim was to address the question if and to what extent regulation by chromatin modifiers and transcription factors is connected to orchestrate multicellular development in ascomycetes. Therefore, in this study we analyzed the functions of the conserved developmental transcription factor PRO44 and the histone chaperone ASF1 during fruiting body development in *S. macrospora*. Both proteins are predicted to act on the regulation of gene expression in the nucleus, and mutants in both genes are blocked at the same stage of development. Thus, we started with the hypothesis that PRO44 and ASF1 might be involved in similar aspects of transcriptional regulation.

## Results

### PRO44 localizes to the nucleus and is strongly expressed in the outer layers of protoperithecia

In a previous study, the *S. macrospora* ASF1 was already shown to localize to the nucleus and to interact with histones H3 and H4 [37], whereas the subcellular localization of PRO44 has not yet been determined. To learn more about the localization and molecular function of PRO44, we first generated a *pro44* deletion mutant by homologous recombination (Additional file 1: Figure S1), since the previously characterized pro44 mutant carries only a point mutation in the stop codon of *pro44* [21]. The Δpro44 mutant shows the same phenotype as the original pro44 mutant carrying the point mutation, namely a developmental block at the stage of protoperithecia formation, and the formation of protoperithecia that are submerged in the agar, in contrast to the wild type, which forms protoperithecia at the agar/air interface. The mutant can be complemented to fertility by transformation with the wild type *pro44* gene (Additional file 1: Figure S1).

**Fig. 1:**
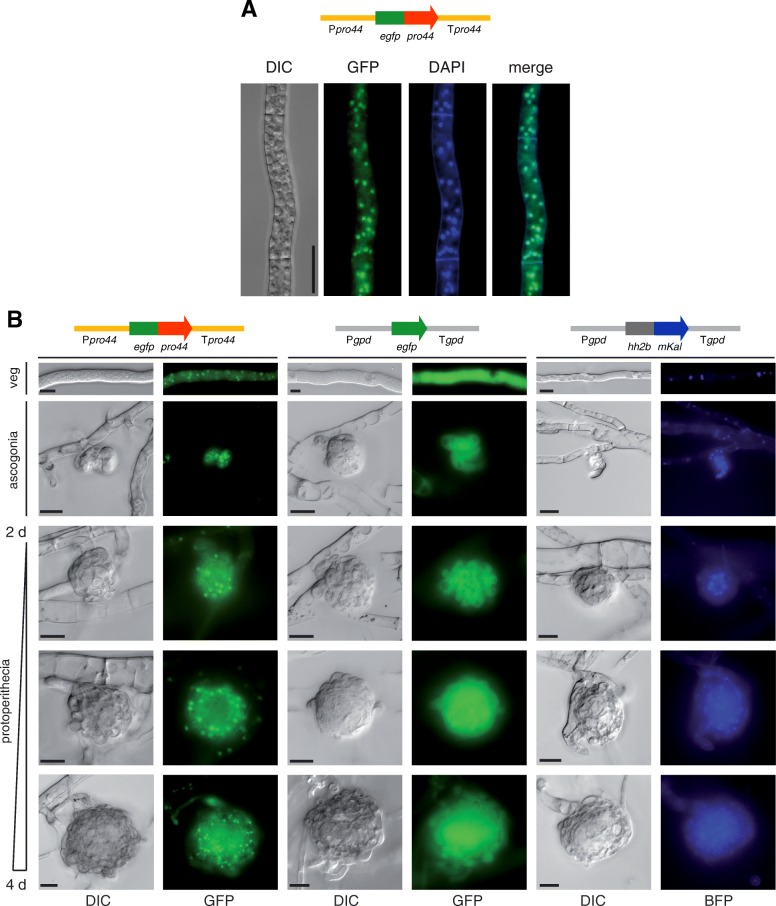
Localization of PRO44. **a** PRO44 localizes to the nucleus. Δpro44 was complemented with an EGFP-PRO44 fusion under control of *pro44* upstream and downstream regions (left, strain S198) and analyzed by fluorescence microscopy. Nuclei were co-stained with DAPI. Scale bar indicates 20 μm. **b** PRO44 localizes to the outer layers of developing fruiting bodies. Strains S198 (left) and T8.1 (middle and right) were analyzed for EGFP fluorescence (left and middle) or H2B-mKalama1 fluorescence (right). Fluorescence was analyzed during a time course of development from vegetative hyphae to young fruiting bodies. Scale bar indicates 10 μm

To analyze the subcellular localization of PRO44, the Δpro44 deletion mutant was transformed with a plasmid expressing an *egfp-pro44* fusion gene under control of the *pro44* 5′ and 3′ regulatory regions. This resulted in fertile transformants confirming complementation of the sterile phenotype by the *egfp-pro44* fusion gene. EGFP fluorescence in hyphae localized in structures stained by DAPI, indicating a nuclear localization of PRO44 as expected for a transcription factor (Fig. 1a).

In a previous analysis, it was found that the *pro44* transcript is strongly expressed in protoperithecia [28]. To check whether a PRO44 accumulation in protoperithecia can also be observed at the protein level, we analyzed the localization of the EGFP-PRO44 fusion protein over a developmental time course (Fig. 1b). As control, a wild type transformant expressing *egfp* from a constitutive promoter as well as an mKalama-fused histone H2B gene was also analyzed. As expected, the wild type control expressing *egfp* alone showed evenly distributed green fluorescence in all mycelial structures. The *mKalama-hh2b*-carrying transformant showed blue nuclear fluorescence in all structures, with a stronger fluorescence in the core of developing protoperithecia than in the outer layers. PRO44 was also detected in nuclei of all structures, with strong fluorescence in developing protoperithecia (Fig. 1b). Interestingly, EGFP-PRO44 fluorescence was stronger in the outer layers of developing protoperithecia than in their core. One possibility for such a distribution of fluorescence would be that the core of the spherical protoperithecia was less accessible for fluorescence microscopy, however, the stronger fluorescence of the mKalama-HH2B fusion in the protoperithecial core of the controls indicates that lack of fluorescence from the protoperithecial core in the case of EGFP-PRO44 is not an artefact. Thus, our data indicate that *pro44* is strongly expressed in developing protoperithecia not only at the transcript, but also at the protein level, and that PRO44 accumulates most strongly in the outer layers of young fruiting bodies.

### PRO44 forms homodimers

**Fig. 2:**
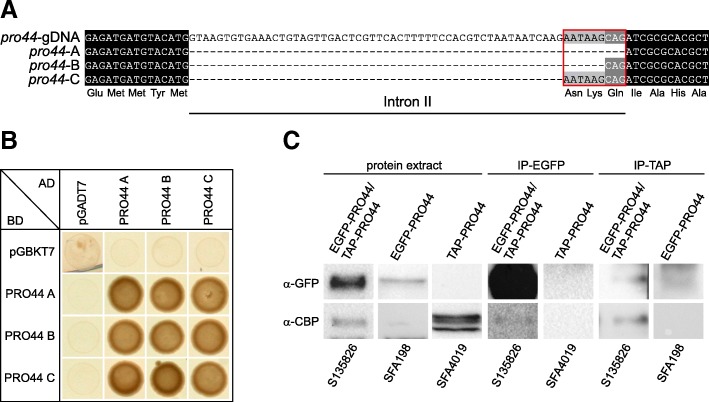
PRO44 forms homodimers. **a** Multiple alignment of the genomic region around the second intron in *pro44*. The three splice variants of *pro44* (*pro44*-A, -B, and -C) are shown. **b** Yeast-two-hybrid analysis of interactions of different PRO44 variants resulting from different splice variants. **c** Co-immunoprecipitation of two differently tagged PRO44 proteins (EGFP- and TAP-tagged proteins in strain S135826, only EGFP-PRO44 in strain SFA198, only TAP-PRO44 in strain SFA4019). The band seen in strain SFA198 (EGFP-PRO44) after TAP-IP and western blot with an anti-EGFP antibody is unspecific (for details see Additional file 2: Figure S2)

To identify PRO44 interaction partners in *S. macrospora*, we performed a yeast two-hybrid screen against two *S. macrospora* cDNA libraries that were established previously [42]. Of several putative interaction partners identified in the screen, only PRO44 itself could be confirmed by targeted two-hybrid analyses with full-length cDNAs, suggesting that PRO44 forms homodimers (Fig. 2). Interestingly, during the cloning of cDNA constructs for the two-hybrid analyses based on RNA from mycelia grown for 4 d in fructification medium, we detected three different splice variants of the second intron of *pro44* (Fig. 2a). One splice variant leads to an additional glutamine in the corresponding protein (PRO44-B), whereas the second leads to the addition of the three amino acids asparagine, lysine, and glutamine (PRO44-C). Structure predictions using Phyre2 [43] indicated an alpha-helical structure in this region for all three proteins. All proteins derived from the splice variants interact with each other in all possible combinations (Fig. 2b); therefore, it is not clear if the different proteins have different biological functions.

To confirm the interaction of PRO44 with itself, we performed chromatin immunoprecipitation with two differently tagged PRO44 variants, one with a TAP (tandem affinity purification)-tag, the other with an EGFP tag (Fig. 2c, Additional file 2: Figure S2). Chromatin immunoprecipitation targeting EGFP or the TAP-tag, respectively, was able to pull down both forms of PRO44, confirming the presence of both tagged PRO44 forms in the same protein complex. These data indicate that PRO44 acts (at least) as a homodimer, although larger protein complexes containing PRO44 cannot be excluded.

### *asf1* plays a role in sexual development, whereas *cac2*, *rtt106*, and *crc1* are not essential for this process

The *S. macrospora* ASF1 protein was shown to localize to the nucleus and to interact with histones H3 and H4, and the *asf1* gene is essential for sexual development [37]. Interestingly, mutants of two other histone chaperone genes for histones H3 and H4, *rtt106* and *cac2*, did not display any developmental phenotype. This might suggest that the developmental function of *asf1* is specific and not generally an effect of chromatin modifier mutations [37]. Therefore, we wanted to address two questions, namely if other chromatin modifier genes that are transcriptionally upregulated during sexual development are also required for this process, and if the nuclear factors ASF1 and PRO44 are involved in regulating the expression of a similar set of genes.

To address the first question, we generated a Δcac2/Δrtt106 double mutant (Additional file 3: Figure S3), as well as a novel deletion mutant of the putative chromatin modifier gene *SMAC_02795* (*crc1*, *crc domain protein 1*, Additional file 4: Figure S4), and double mutants of *crc1* with the three chromatin modifier genes *asf1*, *cac2*, and *rtt106* (Additional file 5: Figure S5). The *crc1* gene was chosen because it is upregulated in protoperithecia of the *S. macrospora* wild type, where it is among the 500 most strongly expressed genes [28]. It encodes a CRC (chromatin remodeling complex) subunit domain protein similar to its *S. cerevisiae* homologs Rsc7 and Swp82. In yeast, Rsc7 and Swp82 are subunits of the chromatin remodeling complexes RSC and SWI/SNF, respectively [44]. These protein complexes are involved in generating nucleosome-free regions upstream of transcribed genes, thereby enabling transcription [45]. In filamentous ascomycetes, two paralogs of Rsc7 and Swp82 can be found; however, at least CRC1 is not clearly orthologous to either of the two proteins (Additional file 6: Figure S6), therefore it was named differently (*crc1* after its characteristic domain). EGFP fusions of the three proteins CAC2, CRC1, and RTT106 localize to the nucleus, as one would expect for (putative) chromatin modifiers (Additional file 7: Figure S7).

When grown on fructification medium (BMM), Δcrc1 is fertile indicating that this gene is not essential for fruiting body formation. To test the degree of redundancy of different chromatin modifier systems with respect to sexual development, we generated double mutants. The double mutants Δcrc1/Δcac2, Δcrc1/Δrtt106, and Δcac2/Δrtt106 are fertile (Additional file 8: Figure S8A). However, the Δcac2/Δrtt106 mutant is sterile when grown on minimal medium, whereas the single mutants Δcac2 and Δrtt106 are fertile under these conditions (Additional file 8: Figure S8B). This result suggests that *cac2* and *rtt106* might have partially redundant functions, and that these functions might be more important under stress conditions like nutrient deprivation. However, loss of both genes does not generally lead to a block in fruiting body formation as the double mutant is fertile on the more nutrient-rich BMM medium.

With respect to double mutants with Δasf1, we were able to generate a Δcrc1/Δasf1 double mutant, which is sterile, similar to the Δasf1 single mutant (Additional file 8: Figure S8). We were not able to generate double mutants of *asf1* and *cac2* or *rtt106* by genetic crossing, suggesting that a combination of these deletions might be lethal. This might suggest that *cac2* and *rtt106* can perform some functions of *asf1*, but not those related to fruiting body formation.

So far, the analysis of chromatin modifier genes *asf1*, *cac2*, *crc1*, and *rtt106* revealed that only *asf1* is essential for fruiting body formation, whereas *cac2* and *rtt106* might have redundant functions in this process under nutrient-limiting conditions.

### RNA-seq analysis reveals distinct roles for *asf1* and *pro44* in sexual development

As ASF1 and PRO44 both are required at the transition from protoperithecia to peritheica, and predicted to influence gene expression, we hypothesized that both genes might be involved in regulating similar subsets of genes. Therefore, we performed RNA-seq to study the functions of both genes with respect to transcription. RNA-seq analyses were carried out with the wild type and the Δasf1 and Δpro44 mutants grown as surface cultures, which leads to sexual development in *S. macrospora*. Furthermore, we analyzed the transcriptome of protoperithecia that were isolated by laser microdissection from the Δpro44 mutant, because *pro44* is preferentially expressed in young fruiting bodies as described above. The Δasf1 mutant did not produce protoperithecia when grown on the slides required for microdissection, therefore no protoperithecial RNA could be isolated from this mutant. The RNA-seq data were analyzed in combination with transcriptome data from protoperithecia of the wild type and developmental mutants pro1 and Δnox1 that were generated previously [28, 46] (Additional file 9: Table S1). The pro1 and Δnox1 mutants are similar to Δpro44 and Δasf1 in that they are only able to form protoperithecia, but no mature perithecia.

**Fig. 3:**
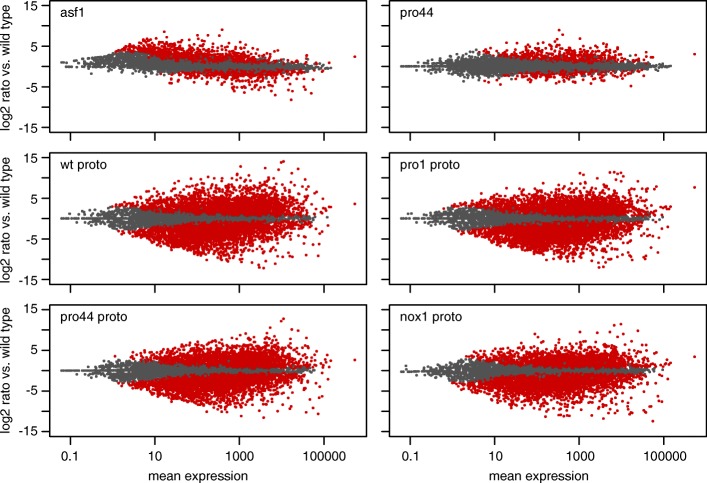
MA plots of expression ratios for six conditions compared to wild type (y-axis) vs. mean expression (x-axis). Ratios and mean expression were calculated with DESeq2. Significantly differentially expressed genes (at padj < 0.1) are shown in red. The following six conditions were compared to wild type sexual mycelium: asf1, sexual mycelium from Δasf1; pro44, sexual mycelium from Δpro44; wt proto, wild type protoperithecia; pro1 proto, protoperithecia from mutant pro1; pro44 proto, protoperithecia from Δpro44; nox1 proto, protoperithecia from Δnox1

When comparing gene expression of all analyzed conditions versus wild type sexual mycelium, it became apparent that the number of differentially regulated genes is much larger when comparing protoperithecia versus sexual mycelium (Fig. 3, middle and bottom row) than in comparisons of sexual mycelium of the mutants Δasf1 or Δpro44 against wild type (Fig. 3, top row). This is consistent with previous findings [28] and confirms that gene expression in young fruiting bodies is vastly different from expression in total sexual mycelium, which in *S. macrospora* consists mostly of vegetative hyphae with the fruiting bodies comprising only a small part of the total bulk of the sexual mycelium. The finding that expression patterns are very different in protoperithecia compared to total sexual mycelium is also supported by an analysis of RPKM (read per kilobase per million counted reads) values, which reflect the overall expression of a gene under the given conditions. Clustering of correlation coefficients based on RPKM values for each sequenced sample clearly shows grouping of the protoperithecia samples apart from the sexual mycelia samples (Additional file 10: Figure S9). Within the protoperithecia samples, the wild type protoperithecia cluster separately from the samples of the three sterile mutants, indicating that there are common expression patterns characteristic of mutants with a block at this developmental stage.

**Fig. 4:**
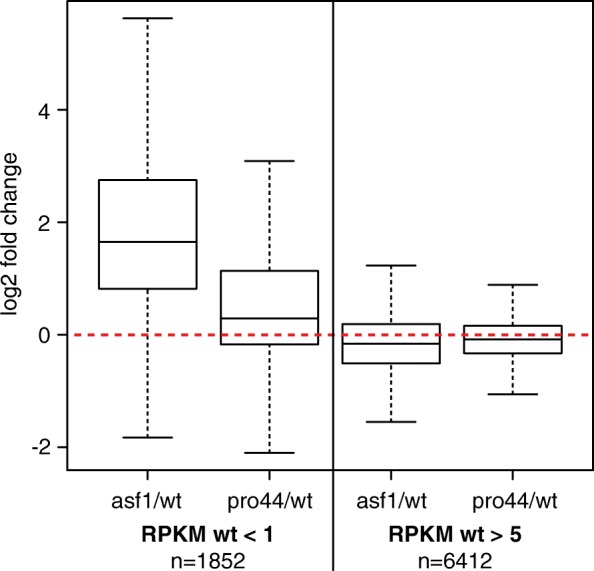
Expression of genes with low RPKM values in the wild type is elevated in the Δasf1 mutant. Box plots of expression ratios (log_2_ of fold change vs. wild type) for genes with RPKM values < 1 or > 5 in the wild type. The box plots show the distribution of fold change values with the median value as a horizontal line in the box between the first and third quartiles. Box plots are given without outliers for better visibility. The red line indicates a log2 fold change of 0 (no change)

Within the sexual mycelia samples, the wild type and Δpro44 cluster together, while the Δasf1 samples cluster separately (Additional file 10: Figure S9). This was somewhat surprising given that the mutants are both sterile with a block at the same developmental stage; however, it might be explained by a distinct tendency of genes with overall low expression to be upregulated specifically in the Δasf1, but not in the Δpro44 mutant (Fig. 3, top row). In all conditions except Δasf1 vs. wild type, the significantly differentially expressed genes are evenly distributed above and below the expression level of the wild type (y = 0 in Fig. 3), and most differentially expressed genes have a medium to high overall expression level (on the x-axis). In Δasf1, however, there is a shift towards the more weakly expressed genes to be upregulated. We confirmed this tendency by analyzing genes that have overall low or high expression in the wild type (RPKM in wild type < 1 or > 5, respectively) for their expression ratios in the two mutants versus the wild type (Fig. 4). The distribution of expression ratios is shifted towards upregulation only in the comparison of Δasf1 versus wild type for genes with overall low expression. Thus, *asf1* appears to be acting as a suppressor of weakly or not expressed genes during sexual development. Overall, the transcriptome analyses show that both *asf1* and *pro44* are required for correct gene expression during sexual development, but that they have different effects on genome-wide patterns of gene expression.

Among the genes that are upregulated in the Δasf1 mutant is *pks4*, a polyketide synthase gene that was previously shown to be essential for fruiting body development [47] (Additional file 11: Figure S10). Expression of *pks4* was verified by RT-qPCR and found to be specifically upregulated in Δasf1 and the Δasf1/Δcrc1 double mutant, but not in mutants of *cac2*, *rtt106*, *pro1* or *pro44* (Additional file 11: Figure S10B). However, the majority of the *pks* and *nrps* genes present in the *S. macrospora* genome is differentially regulated in at least one of the analyzed mutants or conditions (Additional file 11: Figure S10A). A role in fruiting body development has so far been found for *pks4* and *pks7*, the latter being required for melanin biosynthesis in *S. macrospora* [48], but it is tempting to speculate that other secondary metabolism genes that are differentially expressed during development might play a role in this process.

### Nucleosome spacing and positioning around transcriptional start sites are wild type-like in the *asf1* mutant, but cytosine methylation is reduced

The RNA-seq data showed that *asf1* has a very distinct influence on gene expression, which is unique among the *S. macrospora* mutants tested so far. As a histone chaperone, ASF1 is predicted to directly or indirectly influence chromatin structure, and one way for ASF1 to regulate gene expression might be an influence on genome-wide changes of nucleosome positioning, thereby changing the accessibility of promoter regions or gene bodies for transcription. To test this possibility, we performed micrococcal nuclease (MNase)-sequencing with sexual mycelia of the wild type and the Δasf1 mutant. MNase preferentially digests linker DNA between nucleosomes. The remaining nucleosome-bound DNA can be isolated and sequenced [49]. The analysis of MNase-seq data from two independent biological replicates for each strain revealed that the distribution of distances between adjacent nucleosome pairs in the *asf1* mutant does not differ from the wild type (Additional file 12: Figure S11). In both strains, the distribution peaks at a nucleosome distance around 180 bp. This is consistent with each nucleosome consisting of ~ 147 bp of DNA wrapped around a histone octamer, and nucleosomes separated by linkers of varying length, usually from 10 to 80 bp [49–51].

**Fig. 5:**
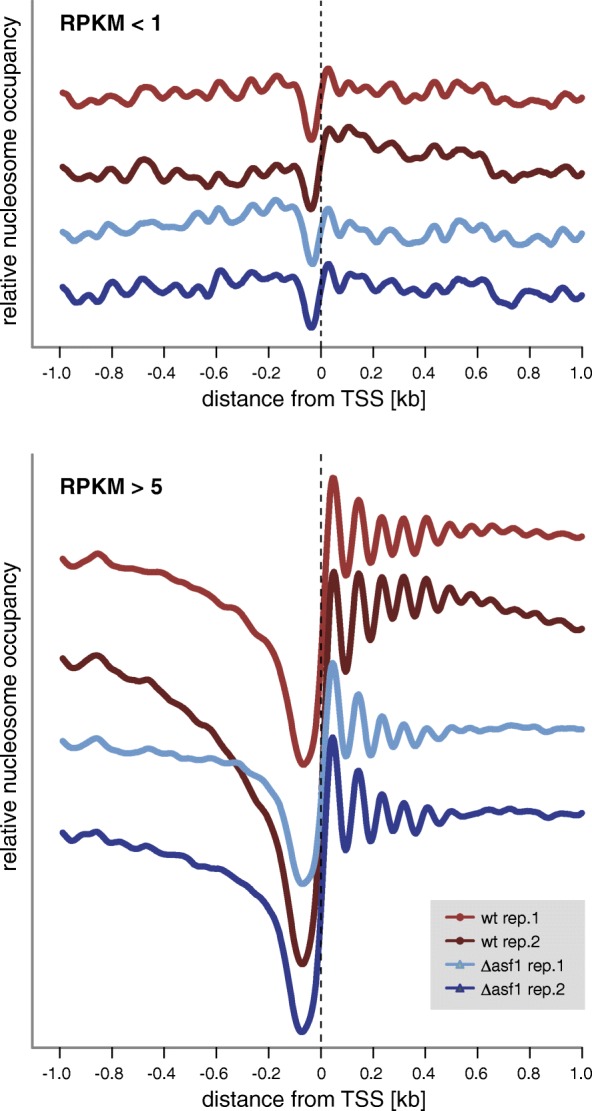
Nucleosome profiles around transcriptional start sites in the wild type and Δasf1. Results are shown for two independent biological replicates for each strain. To account for differences in read coverage, nucleosome occupancy was normalized by setting the highest occupancy for each gene to 1 before calculating averages. Averages at each position were calculated for genes with RPKM values in the wild type of < 1 or > 5, respectively. The y-axis gives the relative nucleosome occupancy within each sample (the scale of the y-axis is the same within each sample, graphs were stacked along the y-axis for better visibility)

It was shown in yeast and metazoa that nucleosomes are unevenly distributed around transcriptional start sites (TSSs), with a nucleosome-depleted region (NDR) directly upstream of the TSS, and regularly spaced nucleosomes downstream of the TSS [49, 50, 52–55]. In human cell lines, this pattern of nucleosome distribution is more pronounced in actively transcribed genes [54]. To determine whether similar patterns can be observed in *S. macrospora*, and whether lack of *asf1* leads to different nucleosome patterns, we analyzed nucleosome positioning around TSS in genes (Fig. 5). For this analysis, we differentiated between genes with overall weak or strong expression (RPKM in wild type < 1 or > 5, respectively), as these are genes that show different degrees of differential gene expression in the Δasf1 strain as described above. NDRs upstream of TSSs were observed in both the wild type and the Δasf1 strain, and in both strains, the NDRs were larger in the more strongly expressed genes (Fig. 5). Thus, the nucleosome distribution around the TSSs is correlated with gene expression levels in *S. macrospora*, similar to findings in other eukaryotes. There was no overall difference in the nucleosome distribution around the TSSs between the wild type and Δasf1. Together with the analysis of nucleosome distances (Additional file 12: Figure S11), this indicates that there are no significant differences in nucleosome number, spacing, and general distribution around TSSs in the *asf1* mutant strain. This indicates that overall nucleosome positioning is not affected by the lack of *asf1*. However, this still leaves other aspects of nucleosome metabolism that might be different in the Δasf1 strain and were not measured in the MNase-seq experiments, e.g. short-term nucleosome positioning at individual genes, nucleosome turnover, or histone modifications.

**Fig. 6:**
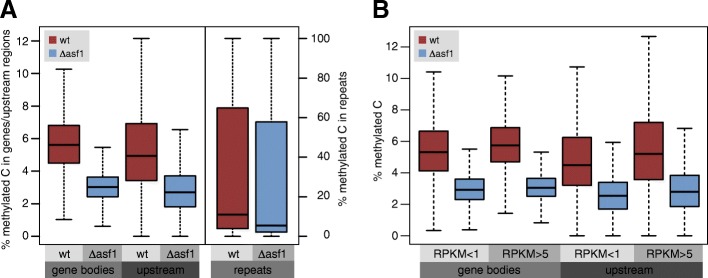
Analysis of cytosine methylation by bisulfite sequencing (BS-seq) in the wild type and Δasf1. **a** Methylated cytosines (at least 5% methylation for a cytosine to be counted as methylated) were counted within gene bodies (exons and introns of gene, i.e. the regions contained in the primary transcript), upstream regions (500 bp upstream of the annotated gene body), and annotated repeat regions. The boxplots show the distribution of the percentages of methylated cytosines, with median value as a horizontal line in the box between the first and third quartiles. Outliers were left out for better visibility. Student‘s t-test showed that mean values of wild type and Δasf1 are significantly different from each other for each group of features (gene bodies, upstream regions, repeats). **b** Boxplot of percentages of methylated cytosines in different subsets of genes. On the left, methylation within gene bodies was analyzed, on the right, methylation in upstream regions, in both cases for genes with RPKM values in the wild type of < 1 or > 5, respectively. Outliers were left out for better visibility. The median value is shown as a horizontal line in the box between the first and third quartiles. Student‘s t-test showed that for each RPKM group (< 1 or > 5), the wild type and Δasf1 mean values are significantly different from each other (*p* < 0.05)

In the yeast *S. cerevisiae*, Asf1 was shown to be involved in regulation of gene expression by histone modifications through recruiting the histone acetyltransferase Rtt109 [38, 56, 57]. The molecular mechanisms of ASF1 action in filamentous fungi have not been analyzed yet. However, histone modifications were analyzed in *N. crassa*, where methylation of lysine 9 of histone H3 was shown to be essential for DNA methylation and heterochromatin formation. In this fungus, H3K9 methylated histone H3 is recognized by heterochromatin protein 1 (HP1), which recruits DNA methyltransferase DIM-2, which is responsible for all cytosine methylation in *N. crassa* [32, 58, 59]. In filamentous fungi, possible connections between histone chaperone functions, histone modifications, and DNA methylation have not yet been studied. To test if *asf1* plays a role in DNA methylation in *S. macrospora*, we performed bisulfite sequencing (BS-seq) for the wild type and the Δasf1 mutant in two independent biological replicates. BS-seq uses sodium bisulfite treatment prior to sequencing to convert cytosines to uracils, whereas methylcytosines remain unmodified. Uracils are read as thymines during sequencing, and by comparing the modified DNA with the reference sequence, the methylation state of the analyzed DNA can be inferred [60, 61]. To address the question whether methylation rates in Δasf1 differ depending on genomic features encoded by the corresponding DNA sequences, we analyzed methylation rates separately in gene bodies (annotated genes including UTRs and introns), upstream regions (500 bp upstream of annotated gene start), and repeat regions (Fig. 6a, Additional file 13: Table S2). Overall methylation rates were higher in repeat regions than in gene bodies and upstream regions, suggesting that DNA methylation might be a mechanism of transposon silencing, similar to what was observed in other ascomycetes [62–64]. Methylation was lower in Δasf1 compared to the wild type in all analyzed types of DNA features (Fig. 6a). This was also the case when looking at those groups of genes that show different expression patterns in the *asf1* mutant, namely genes with overall low or high expression (see above). Both groups of genes showed overall lower methylation rates in Δasf1, both within gene bodies as well as in upstream regions (Fig. 6). Thus, reduced DNA methylation might be involved in the observed changes in gene expression in Δasf1, but additional mechanisms have to be proposed for the effects found specifically for genes with overall low expression.

### *pro44* is required for correct expression of genes in protoperithecia and mycelia

**Fig. 7:**
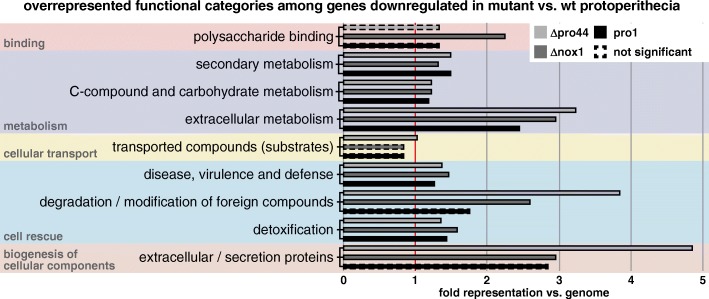
FungiFun2 analysis of genes that are downregulated in mutant protoperithecia compared to wild type protoperithecia. The selected classification ontology was FunCat. Fold representation vs. genome of the first subcategory is indicated in the bar graphs. Main categories to which the subcategories belong are indicated in gray on the left. Only categories are shown that are significantly enriched (*p* < =0.05) in at least one of the mutants

The RNA-seq data for sexual mycelia from *asf1* and *pro44* mutants indicated that both genes are regulating distinct aspects of sexual development as described above. For the Δpro44 mutant, we also generated RNA-seq data for protoperithecia of Δpro44, the last developmental stage that the mutant is able to reach, because the *pro44* gene was previously found to be transcriptionally upregulated in protoperithecia [28], and fluorescence microscopy showed the PRO44 protein to be localized in the outer layers of these structures (Fig. 1). To analyze which groups of genes might be directly or indirectly regulated by *pro44* in protoperithecia, a FungiFun2 analysis based on the FunCat classification was performed for the RNA-seq results [65, 66]. Among the upregulated genes in protoperithecia of any of the three mutants (pro1, Δpro44, or Δnox1), there were no significantly overrepresented FunCat categories; however among the downregulated genes, a number of FunCat categories were significantly overrepresented in at least one of the mutant strains (Fig. 7). Interestingly, in Δpro44 protoperithecia, the categories extracellular metabolism, secretion, and cell rescue were overrepresented among the downregulated genes, indicating that *pro44* is involved in organizing these processes in the developing fruiting body. This fits well with the presence of the protein in the outer layers of the protoperithecium, where cells differentiate to form the perithecium wall, which requires major modifications of the cell wall compared to vegetative cells, and which is the location of first contact for potential predators that feed on fungal fruiting bodies.

Compared to the number of differentially expressed genes in protoperithecia, fewer genes are differentially regulated in sexual mycelia of the Δpro44 mutant. However, among the differentially regulated genes is *cas4*, which is upregulated in Δpro44 even more strongly than in the Δasf1 mutant (Additional file 14: Figure S12). *cas4* is one of four genes encoding carbonic anhydrases in *S. macrospora* [67, 68]. Carbonic anhydrases catalyze the conversion of CO_2_ to bicarbonate (HCO_3_^−^), which is an important metabolite in many physiological reactions. Furthermore, CO_2_ can serve as signaling molecule, e.g. for pathogenic adaptations in *Cryptococcus neoformans* and *Candida albicans* [69]. In *S. macrospora*, three genes (*cas1*, *cas2*, and *cas3*) encode carbonic anhydrases of the β-class that are involved in fruiting body formation and show functional redundancy [70]. The *cas4* gene encodes the only α-class carbonic anhydrase of *S. macrospora*, and deletion of the gene leads to reduced ascospore germination [71]. Interestingly, a quadruple mutant of all four carbonic anhydrase genes forms fruiting bodies within the agar instead of at the agar/air interface [71], and a possible mechanism for involvement of *pro44* and *cas4* in regulating the correct spatial localization of fruiting bodies is discussed below.

### The putative transcription factor gene *asm2* (*SMAC_09436*) is dependent on *pro44* for upregulation in protoperithecia, and is involved in ascospore maturation and discharge

**Fig. 8:**
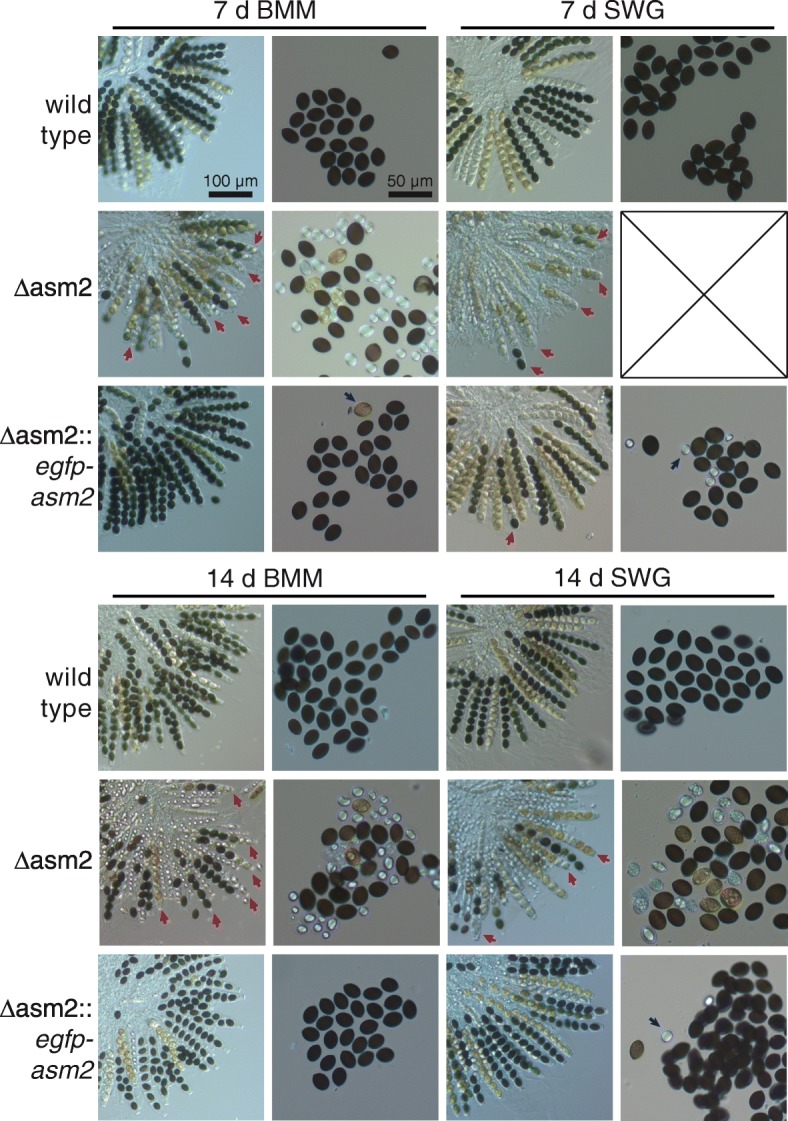
Deletion of the *asm2* (*SMAC_09436*) gene leads to defects in ascospore maturation. Δasm2 (ΔSMAC_09436, spore isolate S148783) and a complemented transformant (spore isolate RL726) were grown on full medium (BMM) or defined medium (SWG) at 25 °C for the indicated times. Scale bars are the same for all ascus rosette pictures (left column for each time point) and pictures of spores discharged on the lid of the petri dish (right column for each time point), respectively. In the *asm2* deletion strain, many ascospores remain less pigmented even after 14 d, and many asci contain mature and immature spores (red arrows), which does not occur in the wild type where spores within an ascus have the same maturation stage. In contrast to the wild type, which only discharges mature (black) ascospores, the deletion mutant discharges immature ascospores. The complemented transformant shows wild type-like ascus and ascospore maturation, and except for few less pigmented spores (blue arrows), only mature spores are discharged

Among the genes that are transcriptionally downregulated in protoperithecia of the *pro44* mutant compared to wild type protoperithecia was *SMAC_09436* (Additional file 9: Table S1). The gene encodes a protein with a GAL4-like zinc cluster domain and a fungal transcription factor domain, and therefore might function as a transcription factor. It has orthologs in other Sordariomycetes, but not outside of this group of fungi (Additional file 15: Figure S13). To determine if *SMAC_09436* is not only regulated by *pro44*, but also involved in fruiting body formation itself, we generated a deletion mutant (Additional file 16: Figure S14). Vegetative growth of the ΔSMAC_09436 mutant was normal, and fruiting bodies were formed after 7 d similar to the wild type. The only difference to the wild type was in the orientation of the fruiting bodies, which in ΔSMAC_09436 was not in all cases perpendicular to the growth medium (Additional file 17: Figure S15). However, the most significant difference to the wild type is a spore maturation defect in the mutant (Fig. 8). In the wild type, the eight spores of an ascus mature at an even rate, i.e. the eight spores within an ascus have the same maturation stage, with the first asci with eight black ascospores visible after 7 d (Fig. 8). In the mutant, the eight ascospores of an ascus showed uneven maturation rates, resulting in asci with mature and immature spores within one ascus, even after prolonged incubation of 14 d (Fig. 8). Further, in contrast to the wild type, where only mature ascospores are discharged, the mutant discharges a mixture of mature and immature ascospores (Fig. 8). Both phenotypes (uneven ascospore maturation and discharge of immature spores) could be complemented by transformation with the wild type *SMAC_09436* gene (Fig. 8). Based on the defect in ascospore maturation, we named the corresponding *SMAC_09436* gene *asm2* (*ascospore maturation 2*) (we did not name it *asm1* to avoid confusion with the *N. crassa asm-1* gene, which is also involved in fruiting body development, but is not a homolog of *SMAC_09436*).

Correct expression of *asm2* in protoperithecia is dependent on *pro44*, and if the *pro44*-dependent transcriptional activation of *asm2* were required for fruiting body formation, any phenotypic defects of Δasm2 should occur in developing fruiting bodies, and at a later stage than the defect in Δpro44. This is consistent with the phenotype of the Δasm2 mutant, which is restricted to ascospore maturation, i.e. in later stages of fruiting body formation than protoperithecial development.

## Discussion

Fruiting body formation in ascomycetes is a complex process involving the concerted actions of many genes. There are a number of transcription factor genes known to be essential for fruiting body development; in addition, several studies in recent years have identified genes that are required for development and encode proteins involved in chromatin modification, pointing to a role for chromatin organization in the spatio-temporal regulation of gene expression during fruiting body morphogenesis [7]. In the basidiomycete *Coprinopsis cinerea*, two recent studies identified the genes *Cc.snf5* and *Cc.apr9* that are required for fruiting body formation [72, 73]. *Cc.snf5* encodes a putative subunit of the SWI/SNF chromatin remodeling complex, while *Cc.arp9* encodes an actin-related protein that is predicted to be a subunit of the SWI/SNF and RSC chromatin remodeling complexes. These data imply a role for chromatin remodeling in fruiting body development of mushrooms. In ascomycetes, two *Fusarium graminearum* homologs of members of the *S. cerevisiae* Set3 complex, HDF1 and FGL1, are required for fruiting body formation. The yeast Set3 complex contains several histone deacetylases (HDAC) and regulates HDAC activity [33, 34]. In *Aspergillus nidulans*, analyses of interaction partners of the velvet protein VeA identified two methyltransferases, VipC and VapB, that can interact with VeA depending on external signals, and inhibit sexual development. Analysis of histone modifications in *vapB* overexpression strains indicated that VapB might be involved in histone H3 lysine 9 trimethylation (H3K9me3) [36]. The VeA protein contains a velvet domain, and velvet domain proteins were only recently shown to be DNA-binding transcription factors [74–76]. Another developmental gene of *A. nidulans*, *rtfA*, was identified as a suppressor of ΔveA secondary metabolism phenotypes, and encodes a putative Paf complex protein [35]. In *S. cerevisiae*, the Paf complex is required for transcription-associated histone modifications [77]. Thus, the physical and genetic interactions of the velvet protein VeA suggest a tightly controlled regulation of specific transcription factors and the chromatin landscape [78].

In this study, we analyzed the role of the chromatin modifier genes *asf1*, *cac2*, *crc1*, and *rtt106* in fruiting body formation in *S. macrospora*. Among these four genes, only *asf1* is essential for this process, while *cac2* and *rtt106* appear to have redundant functions during development under nutrient-limiting conditions. In contrast to several other ascomycetes, which form fruiting bodies only under conditions of nutrient-limination, *S. macrospora* develops fruiting bodies on complete medium [79, 80]. It might be possible that *cac2* and *rtt106* are involved in processes that sense or regulate nutrient availability for the energy-consuming task of perithecium development.

Our analysis of several chromatin modifier genes points to a specific role for *asf1* in fruiting body morphogenesis, and this was confirmed by transcriptome analysis, which revealed an unusually high number of genes with overall low expression being upregulated in the Δasf1 mutant. Thus, *asf1* appears to act as a repressor of genes with low or no expression during fruiting body formation. A repressive role for Asf1 was also recently shown in *S. pombe*, where Asf1 is required for repression of the *fbp1* gene during glucose growth on glucose [81]. The transcriptome pattern observed in the *S. macrospora* Δasf1 mutant is distinct from the pattern observed in the transcription factor mutant Δpro44, which shows an equal number of up- and downregulated genes without a preference for deregulation of genes with low overall expression. Thus, the starting hypothesis that PRO44 and ASF1 might be involved in similar aspects of transcriptional regulation could not be confirmed. While both PRO44 and ASF1 are essential for sexual development and lead to a block of morphogenesis at the stage of protoperithecia, the currently available data indicate that they are involved in distinct aspects of fruiting body formation.

One way for ASF1 to influence transcription would be an effect on nucleosome positioning, especially at TSSs. However, our MNase-seq analyses showed that the distribution of nucleosome pair distances as well as the positioning of nucleosomes around TSSs is not changed in the *asf1* mutant compared to the wild type. By these analyses, we cannot exclude effects of *asf1* on nucleosome turnover as was observed in at the *PHO5* gene promoter in yeast [82–85], or short-lived nucleosome positioning effects, but it is also possible that the effect of *asf1* on transcript levels depends on other chromatin modifications. In yeast, it was shown that Asf1 promotes histone H3 acetylation at lysine 56 by the histone acetyltransferase Rtt109 [57, 86, 87]. However, while a number of proteins involved in histone modifications have been studied in filamentous fungi [29, 88], it is not yet known if ASF1 is required for their function.

In *N. crassa*, histone modification in the form of H3 lysine 9 trimethylation is required for DNA methylation that leads to heterochromatin formation [32]. Thus, a possible linkage between histone chaperone function and changes in gene expression might be in the (indirect) regulation of DNA methylation leading to gene silencing. To test this, we performed BS-seq of the wild type and Δasf1. In the *asf1* mutant, we found a reduction of methylation that was present in all analyzed features (gene bodies, upstream regions and repeat regions). It is possible that the overall lower methylcytosine content in the mutant alters chromatin structure in a way that disturbs normal regulatory events leading to transcriptional expression or silencing of genes. However, another hypothesis would be that ASF1-dependent histone modifications lead to changes in gene expression, and that methylation differences are a consequence rather than a cause of these expression changes. Future studies of histone modifications will be needed to distinguish between these hypotheses.

With respect to the function of chromatin modifiers in cellular differentiation, it was recently shown in mammals that repression of the histone chaperone CAF-1 leads to faster dedifferentiation of somatic cells to stem cells. This is mediated by improved accessibility of chromatin structures at enhancers important for cell fate reprogramming as well as localized changes in methylation of lysine 9 at histone H3, a heterochromatin mark [89, 90]. Therefore, chromatin modifiers might promote developmental transitions by enabling genome-wide changes in chromatin structure that lead to transcriptional reprogramming. Our RNA-seq analysis shows that deletion of *asf1* leads to massive, distinct transcriptome changes, suggesting similar functions for *asf1* in fungi. One possible hypothesis would be that there are several genes essential for downstream developmental processes among the genes activated or repressed by ASF1-mediated chromatin changes. Another hypothesis might be that genome-wide chromatin changes in the absence of *asf1* lead to an overall chromatin state that is sensed by the cells and non-permissive for further development. The nature and function of chromatin changes mediated by ASF1 will be the topic of future studies.

PRO44 belongs to the GATA transcription factors, which constitute a small protein family in filamentous ascomycetes, with only six genes in *N. crassa* [91], all of which have orthologs in *S. macrospora*. In this study, we showed that PRO44 forms homodimers. In filamentous ascomycetes, dimerization between GATA factors was previously shown in several cases, but not for PRO44 orthologs. Known cases include homo- and heterodimerization of the white collar proteins WC-1 and WC-2, which form the white collar complex (WCC) involved in regulating light responses and circadian rhythmicity in *N. crassa* [92, 93]. Another example is the interaction between the GATA factors AreA and AreB in *Fusarium fujikuroi* [94]. Both factors play a role in nitrogen regulation in several ascomycetes [95], although the *N. crassa areB* ortholog *asd4* is involved in sexual development rather than nitrogen regulation, and forms homotetramers [96]. For PRO44, the homodimerization shown in this study is the second type of interaction shown for this protein, the first being an interaction of the PRO44 ortholog SUB-1 with the Zn(II)_2_Cys_6_ transcription factor FF-7 in *N. crassa* [26]. Interestingly, the GATA factor genes *sub-1* and *wc-2* are required for light-dependent eviction of nucleosomes at their DNA binding sites, whereas *ff-7* is not [26]. In *A. nidulans*, the GATA factor AreA is required for chromatin remodeling at the bidirectional promoter of the nitrogen metabolism genes *niiA* and *niaD*, and similar findings were made for a mammalian GATA factor [97–99]. In *N. crassa*, it was shown that histone H3 lysine 14 acetylation at the promoter of the light-inducible *al-3* gene depends on WC-1, which binds to the histone acetyltransferase NGF-1 [100, 101]. The WC-1 homolog LreA of *A. nidulans* interacts with the histone acetyltransferase GcnE and the histone deacetylase HdaA [102]. Thus, GATA factors in fungi may serve as pioneer factors that access nucleosomal DNA and initiate regulatory events including opening of chromatin for other transcription factors, similar to findings in mammals [103]. However, whether PRO44 orthologs have a function in reprogramming chromatin during fruiting body development remains to be elucidated.

In addition to a block at the stage of protoperithecium formation, the Δpro44 mutant is characterized by the formation of protoperithecia that are submerged in the agar instead of differentiating only at the agar/air interface. Thus, *pro44* is required for the correct localization of sexual structures within the mycelium. One possible regulatory pathway that might enable the fungus to sense the agar/air interface might involve the *cas4* gene, which is upregulated in Δpro44 mycelium compared to the wild type. A quadruple mutant of *cas4* and the other three carbonic anhydrase genes in *S. macrospora* was previously shown to have a similar phenotype with submerged perithecia [71]. One might speculate that different CO_2_ concentrations in the agar versus the air act as primary signals for vegetative growth versus sexual development, and that conversion of CO_2_ to bicarbonate by carbonic anhydrases is part of the signaling pathway. If *pro44* acted downstream of this conversion, lack of *pro44* might lead to increased *cas4* expression through a feedback mechanism activated when the signal for elevated CO_2_ concentrations is not received. In addition, lack of signal might lead to mislocalized protoperithecia that are formed in the agar if the signal generated by high CO_2_ concentrations is blocked. Another mutant with a phenotype of submerged perithecia is the adenylyl cyclase mutant Δsac1 [104], therefore it might be hypothesized that a CO_2_-dependent spatial sensing mechanism might involve *pro44*, *sac1*, and carbonic anhydrases. In the pathogenic ascomycete *Candida albicans*, it was shown that CO_2_ acts as a signaling molecule for pathogenic development, and that a carbonic anhydrase and adenylyl cyclase are involved in this process [105, 106]. In the pathogenic basidiomycete *Cryptococcus neoformans*, a carbonic anhydrase was found to mediate the CO_2_-dependent inhibition of cell-cell fusion during sexual development [107]. Thus, it is possible that CO_2_-sensing mechanisms involving carbonic anhydrases and adenylyl cyclases are evolutionary old signaling pathways in fungi that adapted during evolution to mediate different morphogenetic transitions.

Among the genes that are downregulated in Δpro44 protoperithecia compared to wild type protoperithecia are many that are predicted to be involved in secretion, extracellular metabolism, and cell rescue. Combined with the finding that PRO44 is most prominently found in nuclei of cells in the outer layer of the protoperithecium wall, this might indicate that PRO44 is involved in morphogenesis of the rigid perithecium shell. This process requires many tissue differentiations as well as the deposition of extracellular material on the surface of the fruiting body at the protoperithecial-perithecial transition [10]. In a recent study of another sterile mutant, we identified the *spd4* gene, which is also essential for perithecial development. Interestingly, the SPD4 protein localizes to the ascogonial coils within the developing protoperithecium, but not to the outer layers [108]. The molecular function of SPD4 is unknown, as the protein does not have any known functional domains, but future analyses will focus on the roles of differentially expressed genes like *pro44* and *spd4* within the tissues where the corresponding proteins are localized.

Among the genes that are upregulated in wild type protoperithecia compared to mycelium, but downregulated in protoperithecia of the *pro44* mutant compared to wild type protoperithecia is *asm2* (*SMAC_09436*). Generation of a deletion strain and complemented transformants for *asm2* showed that the gene is required for correct ascospore maturation. This phenotype is what one would expect for a gene involved in fruiting body development that is activated by *pro44*, namely being needed later than *pro44* itself, and with a phenotype apparent in the developing fruiting body, where *asm2* is preferentially expressed. Based on the domain architecture of ASM2, the protein is predicted to function as a transcription factor. Thus, the results support the hypothesis that the putative transcription factor ASM2 is acting downstream of PRO44, which itself is dependent on the transcription factor PRO1 for correct expression in protoperithecia [28]. However, based on the available data it is not yet possible to distinguish between a direct and an indirect regulation of *asm2* by PRO44.

## Conclusions

In summary, previous results [28] and the current study indicate that the transcription factors PRO1, PRO44, and ASM2 might act sequentially in a genetic network of transcription factors required for the correct spatio-temporal development of mature fruiting bodies. Based on transcriptome analyses, PRO44 and ASF1 have distinct roles in sexual development, and future analyses will focus on elucidating where chromatin modification and transcriptional regulation meet to orchestrate multicellular development in ascomycetes.

## Methods

### Strains, growth conditions, and genetic crosses

*S. macrospora* strains used in this study are given in Additional File 18: Table S3. Strains were grown in corn meal medium (BMM) or minimal medium (SWG) at 25 °C as described [109, 110]. *S. macrospora* is homothallic (self-fertile), and on BMM or SWG starts to form fruiting bodies at the medium/air interface after three days, maturation is reached after seven days [79]. For RNA extraction from total sexual mycelium for RNA-seq analysis, strains were grown as surface cultures in liquid SWG medium as described [28]. Mycelia harvested after the onset of sexual development contain developing fruiting bodies and the surrounding hyphae, these mycelia are referred to as sexual mycelium. Transformation protocols and protocols for genetic crosses for *S. macrospora* were as described previously [46, 111, 112].

### Cloning procedures

Plasmids for generating gene deletion strains, complementation experiments, fluorescence microscopy or protein interaction studies were cloned by homologous recombination in yeast as described [17]. Oligonucleotides used for generating PCR products for cloning procedures are given in Additional file 19: Table S4, plasmids are given in Additional file 20: Table S5. Deletion cassettes for *pro44*, *crc1*, and *asm2* were generated by amplifying 1 kb genomic regions upstream and downstream of the corresponding genes, which were then cloned to flank the *hph* gene conferring hygromycin resistance [110]. Plasmid pRSnat-pro44-NA contains the *pro44* open reading frame and 1.5 kb upstream and downstream regulatory regions including the 5′ and 3′ untranslated regions (UTRs, approximately 500 bp each), respectively, in pRSnat, which confers nourseothricin resistance in *S. macrospora* [113]. Plasmid pFA20 contains an N-terminal fusion of the *egfp* gene to the *pro44* gene under control of *pro44* upstream and downstream regulatory regions (1.5 kb each, including UTRs). Plasmid pFA30 contains an N-terminal fusion of a TAP (tandem affinity purification)-tag [114, 115] to the *pro44* gene under control of the *Aspergillus nidulans gpd* promoter and *trpC* terminator. Plasmids for yeast two-hybrid analysis containing three different splice variants of *pro44* (A, B, C) were generated by cloning *pro44* cDNAs into yeast vectors pGADT7 and pGBKT7 (Clontech, Palo Alto, CA, USA). Plasmids containing C-terminal fusions of *egfp* to *cac2*, *crc1*, and *rtt106*, respectively, were generated to express the fusion gene under control of the *Aspergillus nidulans gpd* promoter and *trpC* terminator. Plasmid pN_GFP-9436 for complementation of the *asm2* deletion mutant contains an N-terminal fusion of *egfp* to the *asm2* gene under control of the *asm2* upstream and downstream regions (1.6 kb each, including UTRs).

### Generation of gene deletion strains

Deletion strains for *pro44*, *crc1*, and *asm2* were generated by transforming the deletion cassette (upstream and downstream regions flanking the *hph* gene, obtained by restriction digest of the corresponding gene deletion plasmid and gel elution) into a Δku70 strain as described previously [116]. Hygromycin resistant primary transformants were verified for insertion of the deletion cassette by PCR and Southern blot analysis, and knockout strains were crossed against the spore color mutant fus [21] to obtain homokaryotic ascospore isolates carrying the deletion allele in a genetic background without the Δku70 allele.

### Microscopy

For microscopy of mycelia undergoing sexual development, *S. macrospora* strains were grown on glass slides with a thin layer of corn meal extract or corn meal medium solidified with 0.8% agar as described [117, 118]. Fluorescence and light microscopic investigations were carried out with an AxioImager microscope (Zeiss, Jena, Germany). Fluorescence was studied using Chroma (Bellows Falls, VT, USA) filter set 41017 (HQ470/40, HQ525/50, Q495lp) for detection of EGFP, set 49008 (EG560/40x, ET630/75 m, T585lp) for the detection of tdTomato, and set 31000v2 (D350/50, D460/50, 400dclp) for the detection of DAPI and mKalama1. Images were captured with a Photometrix Cool SnapHQ camera (Roper Scientific) and MetaMorph (Universal Imaging). Recorded images were edited with MetaMorph and Adobe Photoshop CS4.

### Yeast two-hybrid analysis

Screening of two yeast two-hybrid libraries and yeast two-hybrid analyses to test for interactions between selected proteins was done as described previously [42].

### Co-immunoprecipitation (co-IP)

Strains carrying plasmid pFA30 (expressing TAP-tagged *pro44*) or pFA20 (expressing *egfp*-tagged *pro44*) were generated by transformation of Δpro44. Fertile transformants were crossed to generate a strain carrying both plasmids. For Co-IP, strains were grown for 3 d at 25 °C in BMM in petri dishes. Harvested, vacuum-dried mycelia were ground in liquid nitrogen. Mycelial powder was centrifuged in extraction buffer (100 mM Tris pH 7.6, 250 mM NaCl, 2 mM EDTA, 10% Glycerin, 0.5% NP-40) for 30 min at 4 °C and 13,500 rpm. Aliquots of the supernatant were used as protein extract for EGFP- or TAP-based immunoprecipitation. For EGFP-based immunoprecipitation (GFP-Trap), 30 μl of GFP beads (ChromoTek GmbH, Planegg-Martinsried, Germany) were centrifuged three times (1 min, 1000 rpm, 4 °C) with 500 μl dilution buffer (10 mM Tris, pH 7.5, 150 mM NaCl, 0.5 mM EDTA, 1 mM PMSF, 0.13 mM benzamidine, 50 μl protease inhibitor cocktail IV/25 ml). 500–600 μl protein extract were added to the GFP beads and incubated for 2 h at 4 °C on a rotation device. Samples were centrifuged (5 min, 5000 rpm, 4 °C), and the supernatant was removed. GFP beads were washed twice with 500 μl dilution buffer. GFP beads were incubated with 80 μl SDS gel loading buffer for 5 min at 100 °C, and the samples were subsequently used for SDS page and Western blotting and immunodetection with anti-GFP monoclonal antibody JL-8 (Clontech, Mountain View, CA, USA). For TAP-tag-based immunoprecipitation, 100 μl IgG beads (GE Healthcare, Freiburg, Germany) were centrifuged (1 min 1000 rpm) in 1 ml TST buffer (50 mM Tris pH 7.6, 150 mM NaCl, 0.05% Tween 20). The supernatant was removed, and 500–600 μl protein extract was added to the beads. Beads were incubated for 3 h at 4 °C on a rotation device, and centrifuged for 1 min at 1000 rpm and 4 °C. Beads were washed twice for 10 min at 4 °C in 1 ml IPP300 buffer (25 mM Tris pH 8, 300 mM NaCl, 0.1% (*w*/*v*) NP-40, 1 mM PMSF, 0.13 mM benzamidine, 2 mM DTT, 50 μl protease inhibitor cocktail IV/25 ml) and pelleted by centrifugation (1 min 1000 rpm). Beads were subsequently washed once in 1 ml IPP150 buffer (25 mM Tris pH 8, 150 mM NaCl, 0.1% (w/v) NP-40, 1 mM PMSF, 0.13 mM benzamidine, 2 mM DTT, 50 μl protease inhibitor cocktail IV/25 ml), and once in 1 ml TEV CB buffer (25 mM Tris/HCl pH 8, 150 mM NaCl, 0.1% NP-40, 0.5 mM EDTA, 1 mM DTT, 1 mM PMSF). Pelleted beads were resuspended in 80 μl SDS gel loading buffer, incubated for 5 min at 100 °C, and the samples were subsequently used for SDS page and Western blotting and immunodetection with anti-Calmodulin Binding Protein Epitope Tag (Millipore, Darmstadt, Germany).

### Laser microdissection

The Δpro44 strain was grown for 4 d at 25 °C on laser microdissection slides from MMI (Molecular Machines and Industries, Zürich, Switzerland), and protoperithecia were isolated with a CellCut Plus system as described [28]. RNA extraction and linear amplification from microdissected samples were as described [28] with the following modification: After addition of extraction buffer, protoperithecia were ground manually in a mortar to improve RNA yield by mechanic destruction of the cell walls. Success of grinding was verified microscopically before proceeding with the RNA extraction.

### RNA extraction and quantitative RT-PCR (RT-qPCR)

Total RNA was prepared as described [119]. Reverse transcription and RT-qPCR were performed as described [47, 120], oligonucleotides used as primers are given in Additional file 19: Table S4. Primer efficiencies were calculated with LinRegPCR [121], and expression ratios were calculated with the ΔΔCt method [122]. The Ct values for an amplicon derived from the SSU rRNA were used as a reference for normalization. Tests for statistical significance were performed with REST [123].

### RNA-seq analysis

RNA-seq analysis of total sexual RNA from the wild type, Δasf1, and Δpro44, as well as amplified RNA from laser microdissected protoperithecia of Δpro44 was done as described previously [28]. For total RNA extraction, strains were grown for 4 d in SWG medium as surface cultures to induce sexual development and allow protoperithecia formation. Mutant strains Δasf1 and Δpro44 have reached their developmental block by this time. Library preparation and sequencing was done at GATC Biotech (Konstanz, Germany). Single reads of 51 nucleotides were obtained for each sample on an Illumina HiSeq2500. For each strain/condition, two independent biological replicates were sequenced. Analysis of RNA-seq data was done as described previously with minor modifications [28]. Briefly, reads were trimmed with custom-made Perl programs to remove reads with nondetermined nucleotides, remove polyA or polyT stretches from end and start of reads, respectively, and trim reads from 3′ and 5′ ends until a base quality of ≥10 was reached. Trimmed reads ≥40 bases were kept for mapping to the *S. macrospora* genome using Tophat v2.2.1 [124, 125]. Reads mapping to annotated features were counted as described [28], and quantitative analysis of gene expression was performed with DESeq2 and LOX [126, 127]. Results of expression analyses for all *S. macrospora* genes are given in Additional file 9: Table S1.

### Micrococcal nuclease sequencing (MNase-seq)

For MNase-seq of the wild type and Δasf1, strains were grown for 3 d in SWG medium as surface cultures. Mycelia were harvested, ground in liquid nitrogen, and suspended in 1 ml MNase buffer (5 mM CaCl_2_, 50 ml Tris-HCl, pH 8) per 100 mg mycelium. 200 μl of resuspended mycelium were treated with 30 units MNase (Thermo Fisher, Waltham, MA, USA) for 10 min at 37 °C with shaking (400 rpm). Samples were centrifuged for 15 min at 15000 rpm, the supernatant was subjected to one round of phenol/chloroform extraction, and treated for 15 min at 37 °C with 10 μl RNase cocktail enzyme mix (Thermo Fisher, Waltham, MA, USA). After another phenol/chloroform extraction step, DNA was precipitated with ethanol, washed and resuspended in 30 μl distilled water. MNase digestion was verified by agarose gel electrophoresis. Library preparation and sequencing was done at GATC Biotech (Konstanz, Germany). Single or paired-end reads were obtained for each sample on an Illumina HiSeq2500. For each strain, two independent biological replicates were sequenced. Reads were trimmed as described [25], and mapped to the *S. macrospora* genome [21, 124] using Bowtie2 v2.2.6 [128]. Resulting .bam files were converted to bed format with BEDTools [129], and nucleosome positions were determined with iNPS v1.2.2 [130]. Further processing of results and analysis of nucleosome distributions around transcriptional start sites (TSS) was done with custom-made Perl scripts using BioPerl [131].

### Bisulfite sequencing (BS-seq)

For BS-seq of the wild type and Δasf1, strains were grown for 4 d in SWG medium as surface cultures. Mycelia were harvested, ground in liquid nitrogen, and suspended in 500 μl lysis buffer (0.6 M NaCl, 10 mM EDTA, 1% SDS, 100 mM Tris-HCl pH 8.0) and 500 μl phenol/chloroform. Samples were subjected to one to two rounds of phenol/chloroform extraction, treated for 60 min at 37 °C with 10 μl RNase cocktail enzyme mix (Thermo Fisher, Waltham, MA, USA), and treated again with phenol/chloroform extraction. The supernatant was cleaned three times on Amicon Ultra 30 k columns (Merck, Darmstadt, Germany) with elution buffer (10 mM Tris-HCl pH 8.0) and eluted in 100 μl of elution buffer. Bisulfite treatment, preparation of a directional library, and sequencing on an Illumina HiSeq were performed at GATC Biotech AG (Konstanz, Germany). Bisulfite conversion rates based on the analysis of the mitochondrial DNA for wild type and Δasf1 were 99.0 and 99.3%, respectively, in the first experiment, and 99.4 and 99.1%, respectively, in the second experiment. Reads were trimmed for low quality bases and adapter removal with Trimmomatic [132] (parameters ILLUMINACLIP:TruSeq3-PE-2.fa:2:30:10:1:TRUE LEADING:3 TRAILING:3 SLIDINGWINDOW:4:15 MINLEN:40). Trimmed reads were mapped to the *S. macrospora* genome and methylated cytosines identified using Bismark [133]. After mapping, reads were deduplicated, and methylated cytosines identified with the Bismark methylation extractor (parameters --ignore_r2 2 --no_overlap). Only cytosines that were methylated in at least 5% of mapped reads were counted as positions where methylation occurred. Further processing of results and analysis of methylation in annotated features (genes, upstream regions and repeat regions) was done with custom-made Perl scripts using BioPerl [131].

## Additional files

Additional file 1: **Figure S1** Generation and complementation of a Δpro44 deletion mutant. A. Overview of the *pro44* genomic locus, the deletion construct is shown above and two plasmids used for generating the probe for Southern blot analysis and for complementing the deletion strain below. B. Southern blot analysis of mutants pro44 and Δpro44 as well as complemented transformants of Δpro44. The Δpro44 strain S106095 was transformed with plasmid pRSnat-pro44-NA, containing *pro44* controlled by its native promoter and terminator regions. Ascospore isolates were obtained from two independent transformants (T99.2 and T99.5), and three single spore isolates from each transformant were subjected to Southern blot analysis after digestion of genomic DNA with *Sal*I. The blot was probed with an 1.5 kb *Bam*HI restriction fragment from pIG3147–1 containing most of the *pro44* gene. The wild type and the pro44 mutant containing a point mutation in *pro44* give a signal at 4.8 kb representing the native *pro44* locus, whereas the deletion mutant does not give a signal as expected. Complemented transformants give a signal at 3.2 kb representing an internal fragment of the native *pro44* locus used for complementation and one or more additional bands indicating at least one plasmid integration event. C. Complementation of Δpro44. Mutant Δpro44 was transformed with plasmid pRSnat-pro44-NA (transformant S108950, Dpro44::SMAC_03223_native). The region of the agar/air interface of longitudinal sections from cultures of the wild type, the sterile mutant pro44, the deletion strain Δpro44 and a complemented transformant of the knockout is shown. The wild type forms perithecia at the surface of the growth medium, while mutants pro44 and Δpro44 only form protoperithecia that are submerged in the agar (white arrowheads point to examples). Complemented transformants are able to produce perithecia at the agar surface like the wild type; however, some protoperithecia and even perithecia are still formed submerged in the medium. (PDF 6507 kb)

Additional file 2: **Figure S2.** Co-immunoprecipitation of strain S198 expressing only *egfp-pro44*, but not *ntap-pro44*. TAP-tag-based immunoprecipitation was performed and the isolated proteins were analyzed by Western blot with an anti-GFP antibody. The part of the Western blot shown in Fig. 2 is labelled in red. Crude extract from the wild type as well as a result from a co-immunoprecipitation of strain S135826 expressing *egfp-pro44* and *ntap-pro44* were used as negative and positive controls, respectively. The unspecific band (green asterisk) seen in the region labelled in red (and in the wild type crude extract, green asterisk) is slightly lower than the EGFP-PRO44 protein detected in the co-IP of strain S135826 (blue asterisk). The Fig. shows a long exposure of the Western Blot detection to better visualize the unspecific bands. (PDF 214 kb)

Additional file 3: **Figure S3.** Southern blot analysis of Δcac2, Δrtt106 double mutants. A. Overview of the *cac2* and *rtt106* genomic loci in the wild type and the corresponding deletion mutants, with the probes for the Southern blot indicated. B. Strains Δrtt106,fus and Δcac2 were crossed and single ascospore isolates were obtained. Three single spore isolates were subjected to Southern blot analysis after digestion of genomic DNA with the indicated enzymes. The blots were probed with the indicated probes. The resulting signals are as expected for the single and double mutants. Triangles indicate the weak bands in two cases, the asterisks in the blot probed with the *rtt106* deletion cassette indicate undigested high molecular weight DNA. (PDF 1231 kb)

Additional file 4: **Figure S4.** Southern blot analysis of Δcrc1 strains. A. Overview of the *crc1* genomic locus in the wild type and the corresponding deletion mutant, with the probes for the Southern blot indicated. B. Southern blot analysis of the wild type, one primary transformant (T124.1) and nine single spore isolates of two different independent primary transformants after digestion of genomic DNA with the indicated enzymes. The blots were probed with the indicated probes. The resulting signals are as expected for the *crc1* deletion for strains T124.1, S123582, S123587, S123615, S123617, S123635, S123637, and S123704 (6.8 kb band when probed with the deletion cassette, and no signal when probed with the gene-specific probe, whereas the wild type and non-deletion-carrying transformants give bands of 3.7 and 4.6 kb when probed with the deletion cassette, and a 8.2 kb band with the gene-specific probe). Deletion strains that were used in further experiments are labelled in red. (PDF 1362 kb)

Additional file 5: **Figure S5.** Southern blot analysis of Δcrc1 double mutants. A. Overview of the genomic locus in the different deletion mutants, with the probe for the Southern blot indicated. b. Southern blot analysis of the wild type and the Δcrc1, Δrtt106 double mutant S126403 after digestion of genomic DNA with HindIII. The blot was probed with the *hph* cassette. The resulting signals are as expected for the double mutant (6.8 kb band for the Δcrc1 locus and 3.7 kb band for the Δrtt106 locus) and the wild type (no signal). C. Southern blot analysis of the wild type and single ascospore isolates from crosses of Δcrc1 and Δasf1, or Δcrc1 and Δcac2, respectively. Genomic DNA was digested with *Hin*dIII. The blot was probed with the *hph* cassette. Double mutants Δcrc1, Δasf1 can be identified by bands at 6.8 kb for the Δcrc1 deletion and 3.5 kb band for the Δasf1 deletion. Double mutants Δcrc1, Δcac2 can be identified by bands at 6.8 kb for the Δcrc1 deletion and 4.2 kb band for the Δcac2 deletion. Double deletion strains that were used in further experiments are labelled in red. (PDF 1061 kb)

Additional file 6: **Figure S6.** Phylogenetic analysis of CRC subunit-containig proteins in ascomycetes. Protein sequences were aligned with Clustal X, and a Neighbor Joining analysis was performed with PAUP* with 1000 bootstrap replicates (bootstrap percentages are given at the branches). Sequences from the following ascomycetes were used for analysis with the CRC-subunit protein CC1G_08669 from the basidiomycete *Coprinopsis cinerea* (CC1G) as an outgroup: *Aspergillus nidulans* (ANID), *Neurospora crassa* (NCU), *Pyronema confluens* (PCON), *Saccharomyces cerevisiae* (S.c.), *Schizosaccharomyces pombe* (S.p.), *Sclerotinia sclerotiorum* (SS1G), *Sordaria macrospora* (SMAC), *Stagonospora nodorum* (SNOT), *Tuber melanosporum* (GSTUMT). The *S. macrospora* CRC1 protein is part of a cluster of proteins on a separate branch from the cluster containing the *S. cerevisiae* and *S. pombe* Rsc7 proteins. (PDF 131 kb)

Additional file 7: **Figure S7.** Analysis of subcellular localization of RTT106, CAC2, and CRC1 by fluorescence microscopy. A. Nuclear localization of RTT106 and CAC2. Both genes were fused with *egfp* and co-transformed with plasmid pRH2B expressing a tdTomato-labelled histone. RTT106 and CAC2 co-localize with the histone in the nucleus. Growth 2d on slides with BMM, scale bar 20 μm. pRH2B contains genes for histone H2B fused with tdTomato (Teichert et al. 2014, PLoS Genet. 10:e1004582). B. Nuclear localization of CRC1. *crc1* fused with *egfp* and expressed from plasmid pSMAC_02795_EGFP. CRC1 co-localizes with the DAPI-labelled nuclei. Growth 2d on slides with BMM, scale bar 10 μm. (PDF 3836 kb)

Additional file 8: **Figure S8.** Fruiting body development in chromatin modifier mutants. A. Strains were grown for 7 d on BMM at 25 °C. Photographs show fruiting body development on petri dishes, small boxes in lower left of each picture show enlarged sections of each overview. Scale bar in small boxes is 2 mm. B. Strains were grown for 7 d on SWG at 25 °C. Photographs show fruiting body development on petri dishes. (PDF 5774 kb)

Additional file 9: **Table S1.** RNA-seq analysis of *S. macrospora* wild type and mutant strains. (XLSX 8905 kb)

Additional file 10: **Figure S9.** Heatmap of Spearman‘s correlation coefficients for pairwise comparisons of the sets of RPKM values (log_2_ transformed) for each analyzed RNA-seq sample (two independent biological replicates per strain/condition). Included were RPKM values for all genes that had a measurement in all samples. Clustering and heatmap generation were done in R. (PDF 161 kb)

Additional file 11: **Figure S10.** Expression of *pks* and *nrps* genes in different mutants and tissues. A. Heatmap was generated based on the log2 ratios from the DESeq2 analysis of RNA-seq data. Two *pks* genes that were previously shown to be involved in fruiting body formation (*pks4* for fruiting body formation and morphology, *pks7* for melanin formation for black pigmentation of perithecia and ascospores) are given in bold. The results show that *pks4* is indeed upregulated in Δasf1, but not Δpro44 mycelium. Furthermore it is upregulated in all protoperithecia samples compared to wild type, again showing that expression is not strongly dependent on *pro44*, *pro1*, or *nox1*. Overall, several *pks* and *nrps* genes are upregulated in Δasf1 mycelia, more than in Δpro44 mycelia, and about half of the genes are strongly upregulated in wild type protoperithecia. B. Expression of *pks4* in transcription factor or chromatin modifier mutants. Quantitative RT-PCR analysis of *pks4* expression was performed, ratios and standard errors (given as error bars) were calculated with REST. Dots indicate significantly differential expression (REST, *p* < =0.05). Data for *pro1* and *pro44* are from Schindler and Nowrousian 2014 (Fungal Genet Biol 68: 48–59). (PDF 199 kb)

Additional file 12: **Figure S11.** Distances between nucleosome pairs as determined by iNPS analysis of MNase-seq reads of wild type and Δasf1. Results are shown for two independent biological replicates for each strain. A. Analyzed were distances between pairs of nucleosomes of the iNPS category “MainPeak”. B. Analyzed were all nucleosome pair distances, irrespective of nucleosome type determined by iNPS (nucleosome types MainPeak, MainPeak:doublet, MainPeak+Shoulder, Shoulder). (PDF 157 kb)

Additional file 13: **Table S2.** Bisulfite sequencing (BS-seq) of the wild type and the *asf1* mutant. (XLSX 1770 kb)

Additional file 14: **Figure S12.** Expression of carbonic anhydrase genes of *S. macrospora* in different mutants/conditions. Hierarchical clustering and heatmap generation of the log2 of fold ratios as determined in the DESeq2 analysis were done in R. (PDF 162 kb)

Additional file 15: **Figure S13.** Multiple alignment of SMAC_09436 (ASM2) orthologs. Orthologs were determined by bidirectional BLASTP analyses. Proteins from the Sordariomycetes *Sordaria macrospora* (*S.m.*, SMAC_09436), *Neurospora crassa* (*N.c.*, NCU010258), *Podospora anserina* (*P.a.*, CDP26737.1), *Magnaporthe oryzae* (*M.o.*, XP_003720415.1), *Fusarium graminearum* (*F.g.*, FGRAMPH1_01T14721), and *Trichoderma reesei* (*T.r.*, XP_006961730.1) were aligned with ClustalX. No clear orthologs outside of the Sordariomycetes could be identified. The GAL4 (GAL4-like Zn2Cys6 binuclear cluster DNA-binding) domain and the fungal_TF_MHR (fungal transcription factor regulatory middle homology region) domain in SMAC_09436 are indicated by black and grey bars, respectively, above the sequence. (PDF 176 kb)

Additional file 16: **Figure S14.** Southern blot/PCR analysis of Δasm2 (ΔSMAC_09436) strains and complemented transformants. A. Overview of the *asm2* genomic locus in the wild type and the corresponding deletion mutant, with the probes for the Southern blot indicated. Below, the *SMAC_09436*-containing region of complementation vector pN_GFP-9436 is shown. Blue and yellow arrows indicate primers for PCRs described in C. B. Southern blot analysis of the wild type and ten single spore isolates after digestion of genomic DNA with *Pst*I. The blots were probed with the indicated probes. The resulting signals are as expected for both probes (sizes indicated below the blots). Deletion strains that were used in further experiments are labelled in red. C. Ectopic integration of complementation plasmid pN_GFP-9436 was confirmed by PCR. Six different single spore isolates from two different complemented transformants were analyzed with two different primer sets (see A). Spore isolates RL726 and RL740 are based on deletion strain S148783 as recipient for transformation, spore isolates RL754, RL756, RL760, and RL775 are based on S148694. As expected, with the primers amplifying a fragment from *egfp* to the *asm2* promoter region (yellow arrows in A), amplicons were obtained only with the complemented transformants and the plasmid control. With primers amplifying a fragment from the C-terminal region to the terminator region of *asm2*, only complemented transformants, the wild type, and the plasmid control gave amplicons as expected. (PDF 1498 kb)

Additional file 17: **Figure S15.** Morphological characterization of Δasm2 (ΔSMAC_09436, spore isolate S148783) and a complemented transformant (spore isolate RL726). Strains were grown on full medium (BMM) or defined medium (SWG) at 25 °C for the indicated times. Scale bars are the same for all overview (left column for each time point, top view of perithecia growing on the surface of the agar medium) and detail (right column for each time point, side view of perithecia growing on the surface of the agar medium) pictures. In the *asm2* deletion strain as well as the complemented transformant, perithecia are sometimes not arranged perpendicular to the growth surface as in the wild type (red arrows). (PDF 5730 kb)

Additional file 18: **Table S3.** *Sordaria macrospora* strains used in this study. (PDF 288 kb)

Additional file 19: **Table S4.** Oligonucleotides used in this study. (PDF 318 kb)

Additional file 20: **Table S5.** Plasmids used in this study. (PDF 270 kb)
