# Supplementary figures and images for: The transcription factor PRO44 and the histone chaperone ASF1 regulate distinct aspects of multicellular development in the filamentous fungus Sordaria macrospora
Source: BMC Genet. 2018 Dec 13;19:112. doi: 10.1186/s12863-018-0702-z (PMC6293562; doi:10.1186/s12863-018-0702-z)

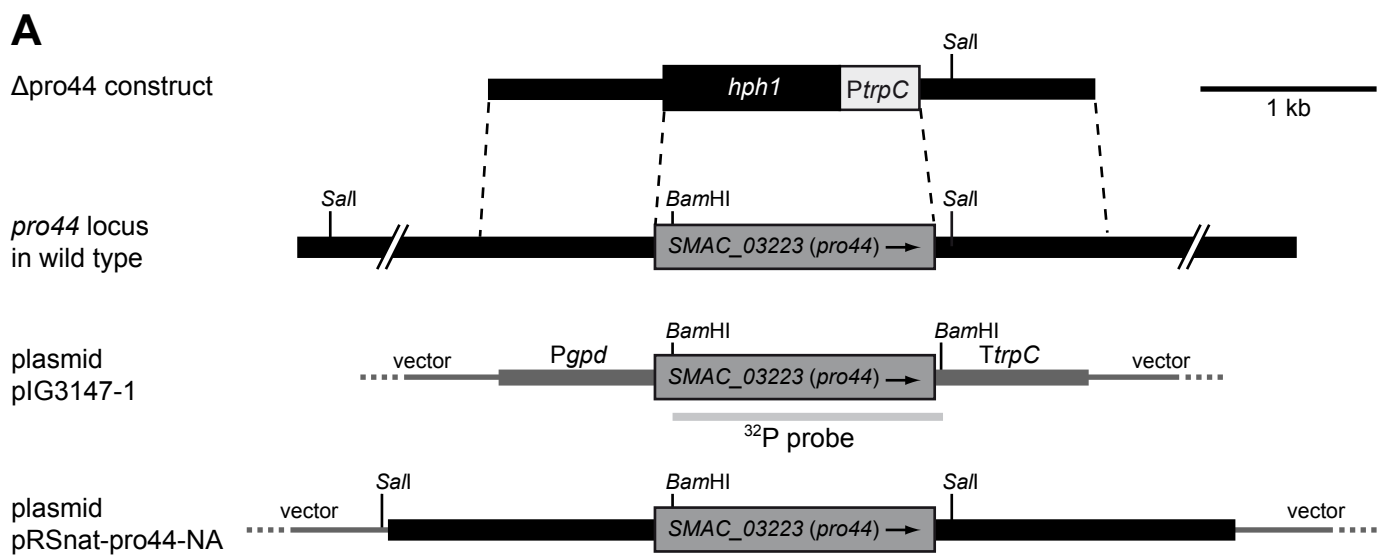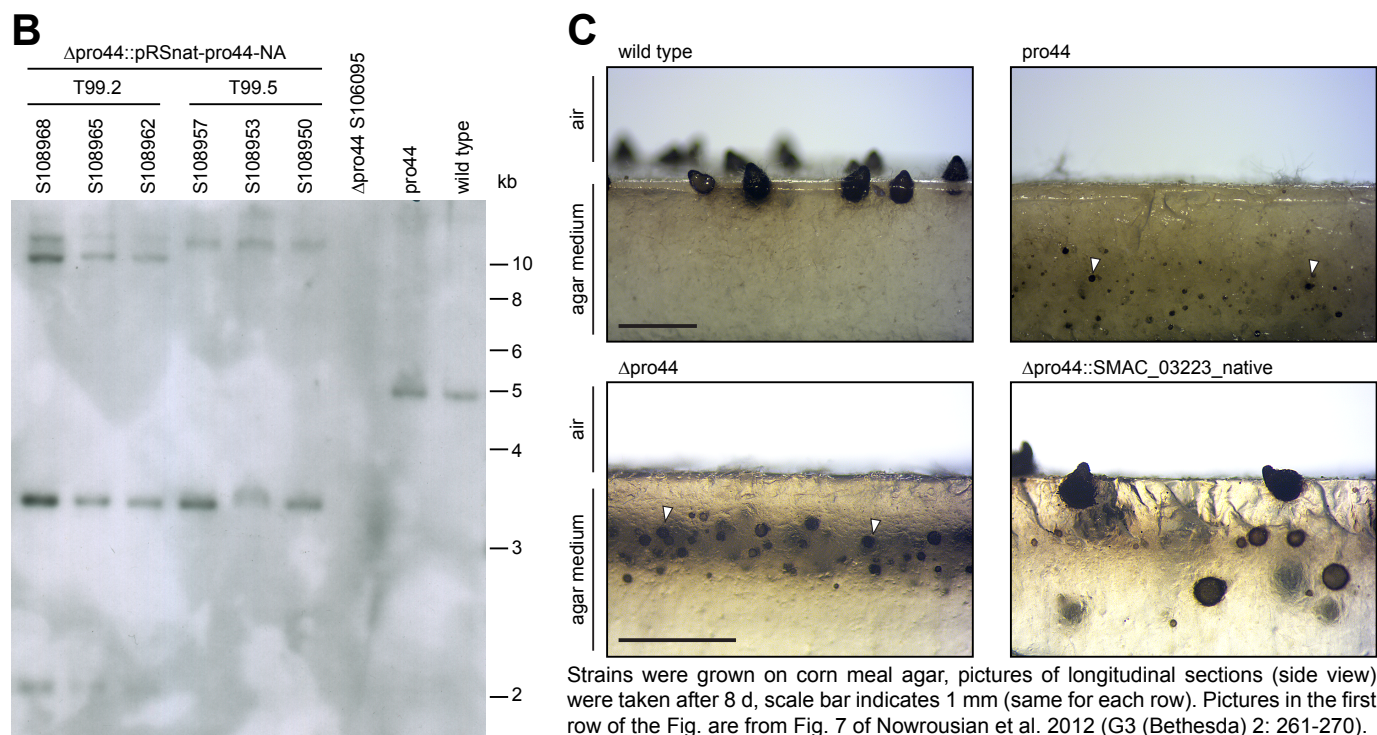

**Figure S1**

Supplement: Supplementary file 1 — Figure S1 Generation and complementation of a Δpro44 deletion mutant. A. Overview of the pro44 genomic locus, the deletion construct is shown above and two plasmids used for generating the probe for Southern blot analysis and for complementing the deletion strain below. B. Southern blot analysis of mutants pro44 and Δpro44 as well as complemented transformants of Δpro44. The Δpro44 strain S106095 was transformed with plasmid pRSnat-pro44-NA, containing pro44 controlled by its native promoter and terminator regions. Ascospore isolates were obtained from two independent transformants (T99.2 and T99.5), and three single spore isolates from each transformant were subjected to Southern blot analysis after digestion of genomic DNA with SalI. The blot was probed with an 1.5 kb BamHI restriction fragment from pIG3147–1 containing most of the pro44 gene. The wild type and the pro44 mutant containing a point mutation in pro44 give a signal at 4.8 kb representing the native pro44 locus, whereas the deletion mutant does not give a signal as expected. Complemented transformants give a signal at 3.2 kb representing an internal fragment of the native pro44 locus used for complementation and one or more additional bands indicating at least one plasmid integration event. C. Complementation of Δpro44. Mutant Δpro44 was transformed with plasmid pRSnat-pro44-NA (transformant S108950, Dpro44::SMAC_03223_native). The region of the agar/air interface of longitudinal sections from cultures of the wild type, the sterile mutant pro44, the deletion strain Δpro44 and a complemented transformant of the knockout is shown. The wild type forms perithecia at the surface of the growth medium, while mutants pro44 and Δpro44 only form protoperithecia that are submerged in the agar (white arrowheads point to examples). Complemented transformants are able to produce perithecia at the agar surface like the wild type; however, some protoperithecia and even perithecia are still formed submerged in the medi [file 12863_2018_702_MOESM1_ESM.pdf]

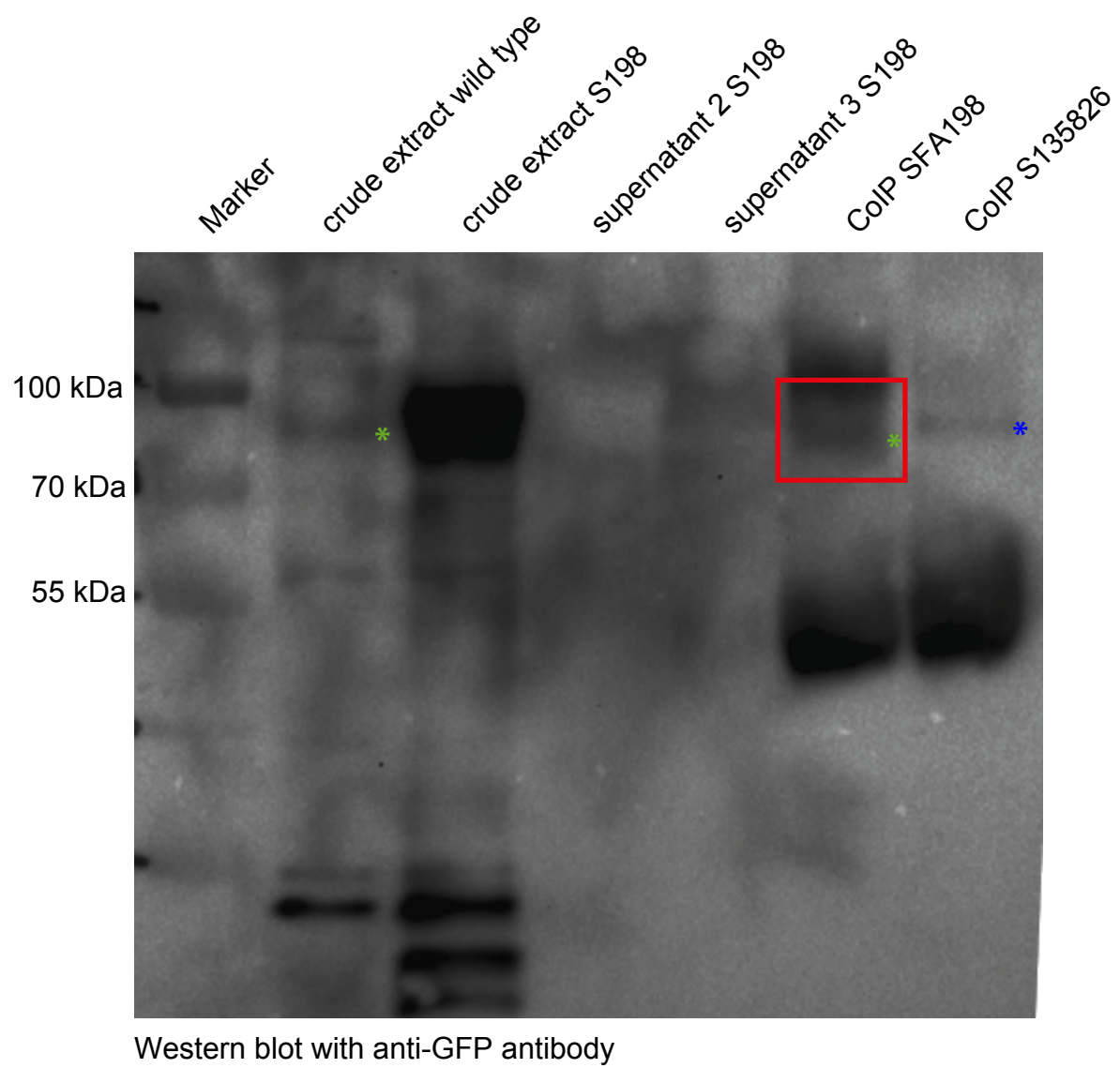

**Figure S2**

Supplement: Supplementary file 2 — Figure S2. Co-immunoprecipitation of strain S198 expressing only egfp-pro44, but not ntap-pro44. TAP-tag-based immunoprecipitation was performed and the isolated proteins were analyzed by Western blot with an anti-GFP antibody. The part of the Western blot shown in Fig. 2 is labelled in red. Crude extract from the wild type as well as a result from a co-immunoprecipitation of strain S135826 expressing egfp-pro44 and ntap-pro44 were used as negative and positive controls, respectively. The unspecific band (green asterisk) seen in the region labelled in red (and in the wild type crude extract, green asterisk) is slightly lower than the EGFP-PRO44 protein detected in the co-IP of strain S135826 (blue asterisk). The Fig. shows a long exposure of the Western Blot detection to better visualize the unspecific bands. (PDF 214 kb) [file 12863_2018_702_MOESM2_ESM.pdf]

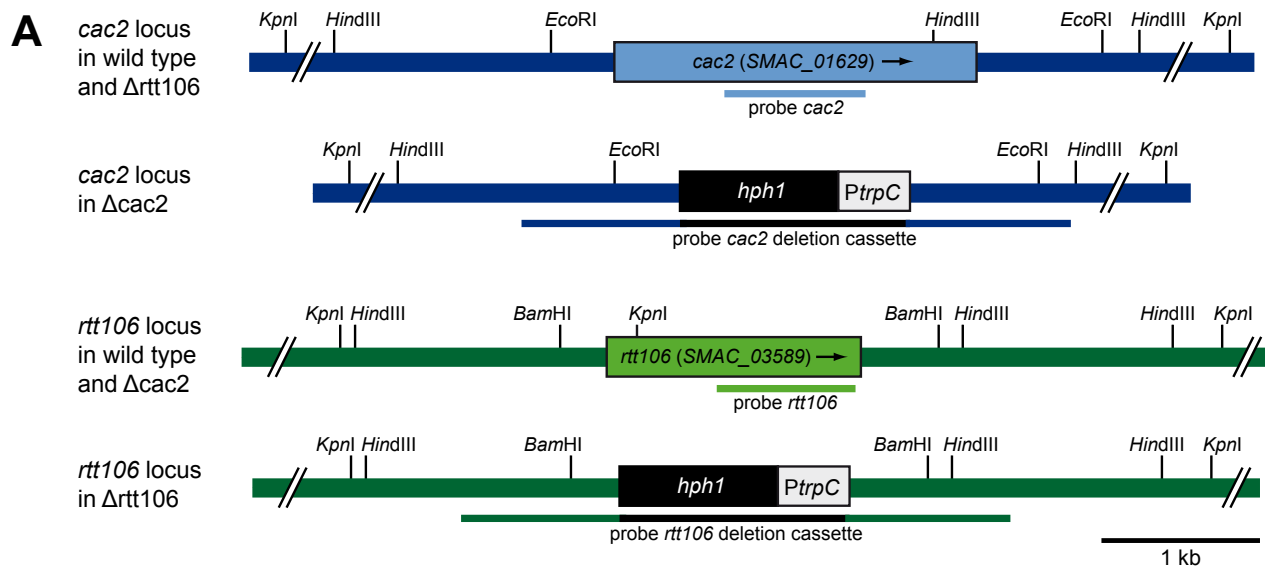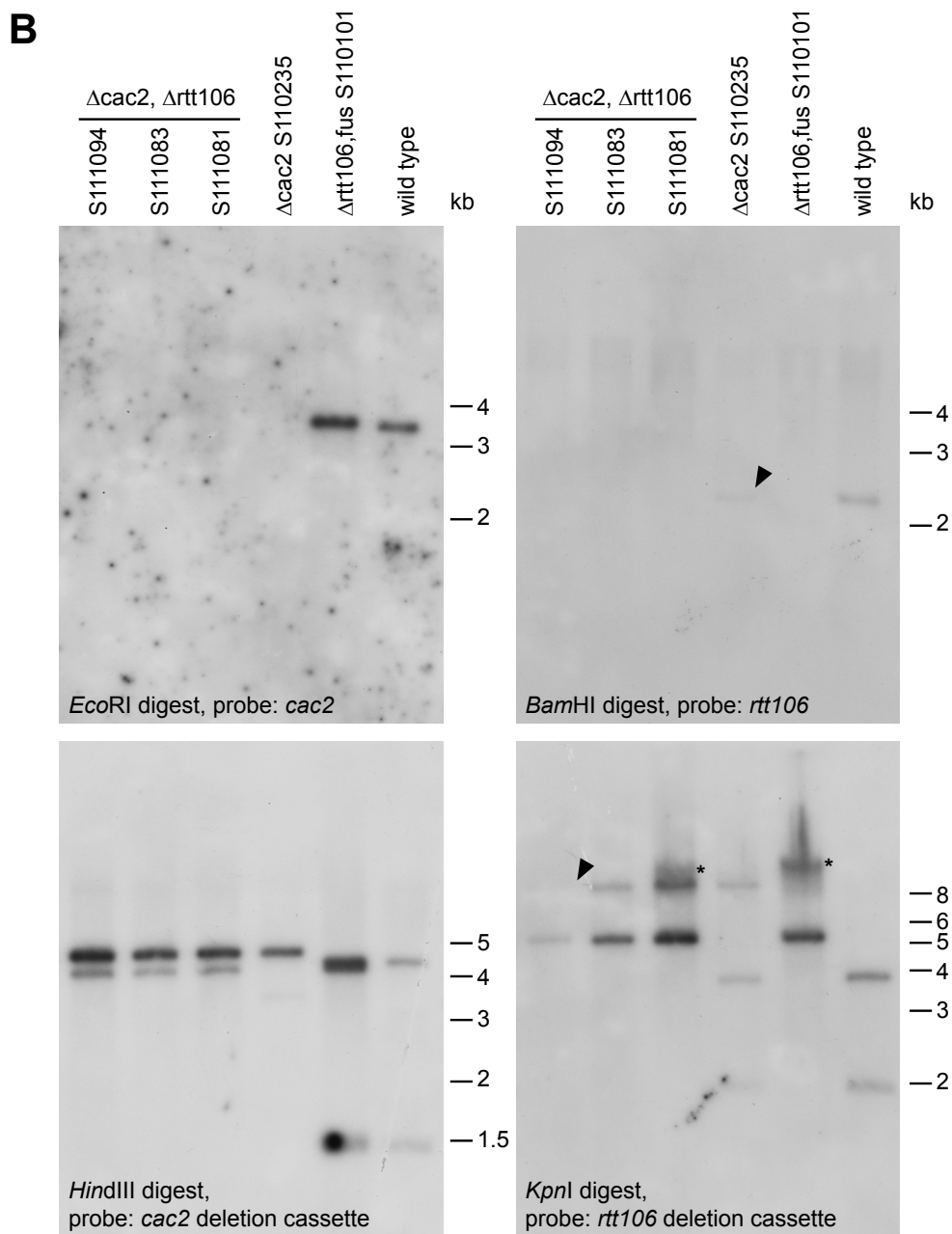

Figure S3

Supplement: Supplementary file 3 — Figure S3. Southern blot analysis of Δcac2, Δrtt106 double mutants. A. Overview of the cac2 and rtt106 genomic loci in the wild type and the corresponding deletion mutants, with the probes for the Southern blot indicated. B. Strains Δrtt106,fus and Δcac2 were crossed and single ascospore isolates were obtained. Three single spore isolates were subjected to Southern blot analysis after digestion of genomic DNA with the indicated enzymes. The blots were probed with the indicated probes. The resulting signals are as expected for the single and double mutants. Triangles indicate the weak bands in two cases, the asterisks in the blot probed with the rtt106 deletion cassette indicate undigested high molecular weight DNA. (PDF 1231 kb) [file 12863_2018_702_MOESM3_ESM.pdf]

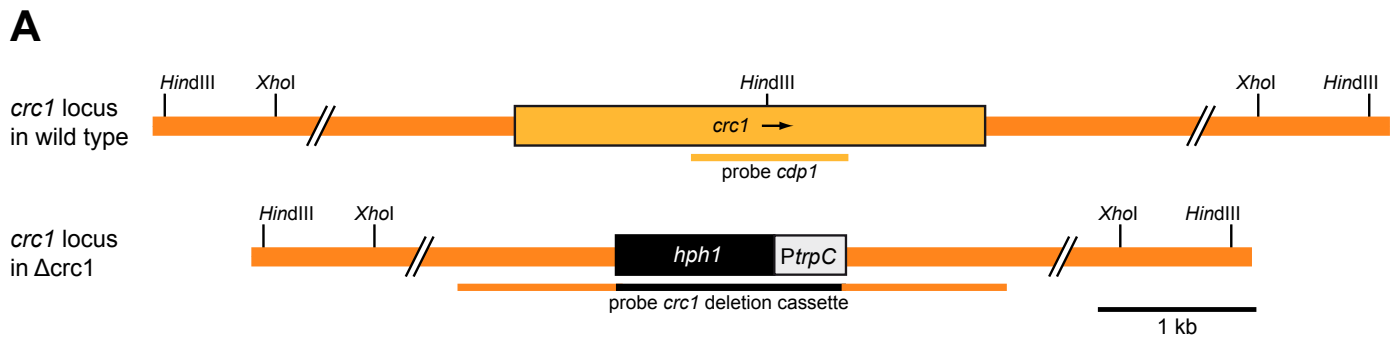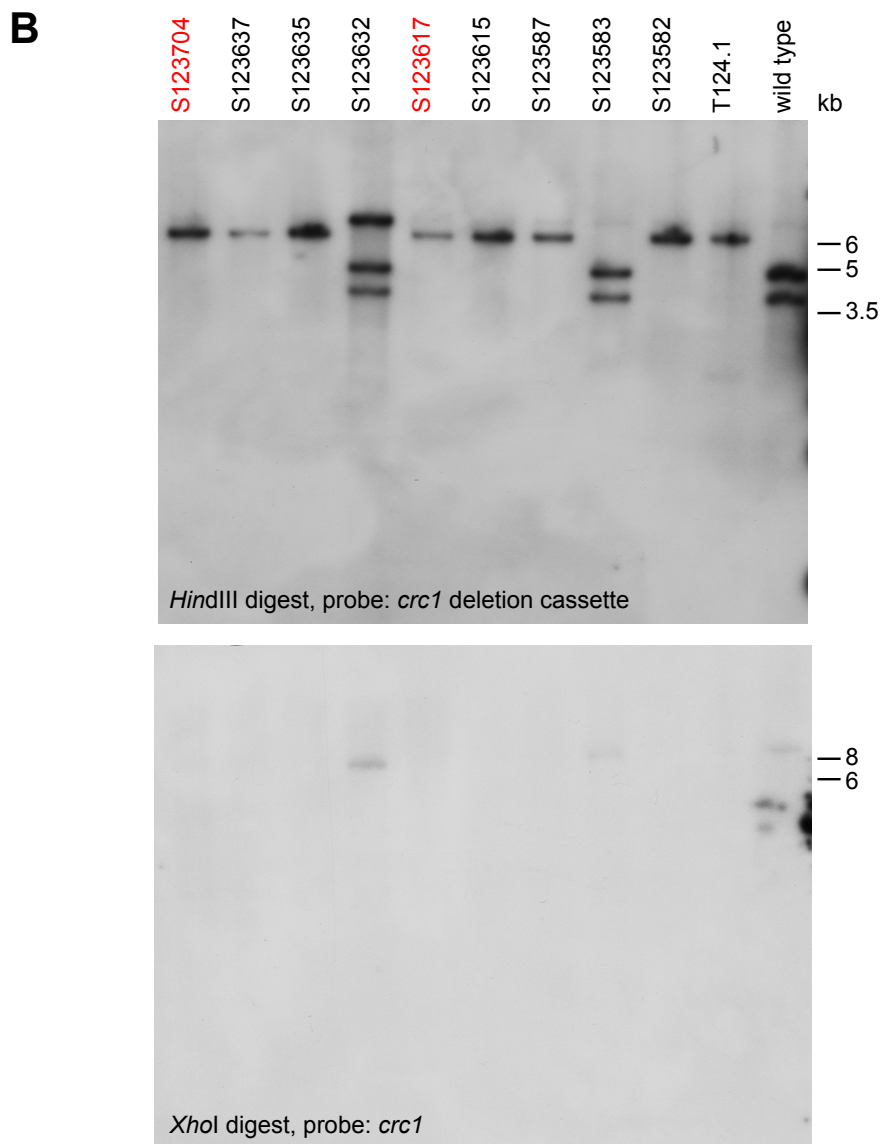

**Figure S4**

Supplement: Supplementary file 4 — Figure S4. Southern blot analysis of Δcrc1 strains. A. Overview of the crc1 genomic locus in the wild type and the corresponding deletion mutant, with the probes for the Southern blot indicated. B. Southern blot analysis of the wild type, one primary transformant (T124.1) and nine single spore isolates of two different independent primary transformants after digestion of genomic DNA with the indicated enzymes. The blots were probed with the indicated probes. The resulting signals are as expected for the crc1 deletion for strains T124.1, S123582, S123587, S123615, S123617, S123635, S123637, and S123704 (6.8 kb band when probed with the deletion cassette, and no signal when probed with the gene-specific probe, whereas the wild type and non-deletion-carrying transformants give bands of 3.7 and 4.6 kb when probed with the deletion cassette, and a 8.2 kb band with the gene-specific probe). Deletion strains that were used in further experiments are labelled in red. (PDF 1362 kb) [file 12863_2018_702_MOESM4_ESM.pdf]

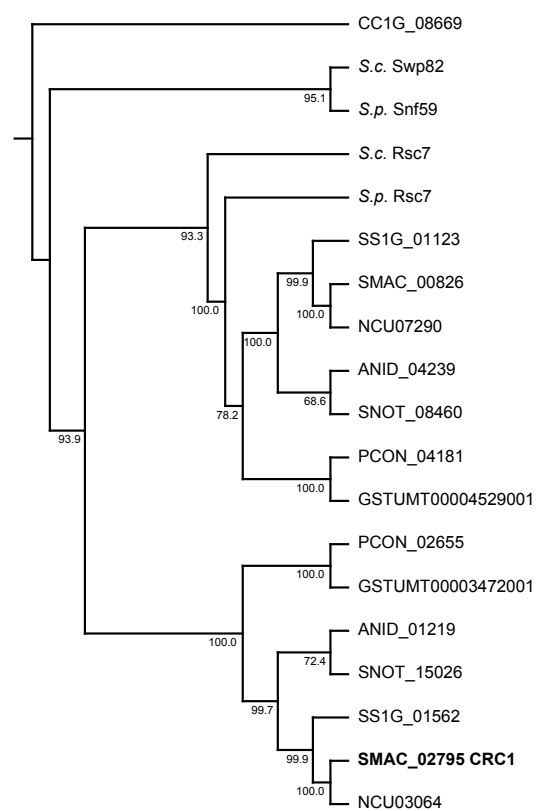

**Figure S6**

Supplement: Supplementary file 6 — Figure S6. Phylogenetic analysis of CRC subunit-containig proteins in ascomycetes. Protein sequences were aligned with Clustal X, and a Neighbor Joining analysis was performed with PAUP* with 1000 bootstrap replicates (bootstrap percentages are given at the branches). Sequences from the following ascomycetes were used for analysis with the CRC-subunit protein CC1G_08669 from the basidiomycete Coprinopsis cinerea (CC1G) as an outgroup: Aspergillus nidulans (ANID), Neurospora crassa (NCU), Pyronema confluens (PCON), Saccharomyces cerevisiae (S.c.), Schizosaccharomyces pombe (S.p.), Sclerotinia sclerotiorum (SS1G), Sordaria macrospora (SMAC), Stagonospora nodorum (SNOT), Tuber melanosporum (GSTUMT). The S. macrospora CRC1 protein is part of a cluster of proteins on a separate branch from the cluster containing the S. cerevisiae and S. pombe Rsc7 proteins. (PDF 131 kb) [file 12863_2018_702_MOESM6_ESM.pdf]

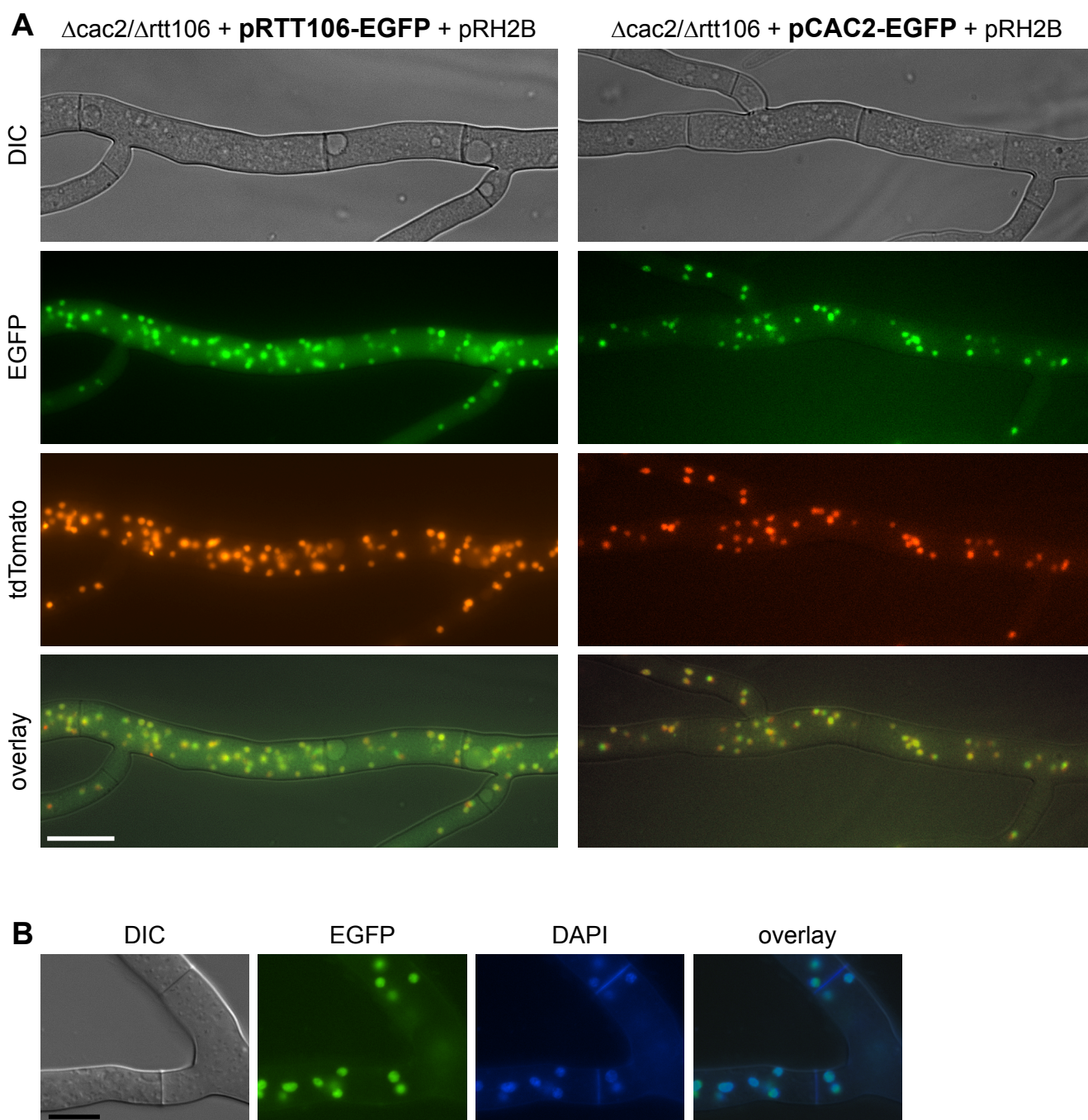

**Figure S7**

Supplement: Supplementary file 7 — Figure S7. Analysis of subcellular localization of RTT106, CAC2, and CRC1 by fluorescence microscopy. A. Nuclear localization of RTT106 and CAC2. Both genes were fused with egfp and co-transformed with plasmid pRH2B expressing a tdTomato-labelled histone. RTT106 and CAC2 co-localize with the histone in the nucleus. Growth 2d on slides with BMM, scale bar 20 μm. pRH2B contains genes for histone H2B fused with tdTomato (Teichert et al. 2014, PLoS Genet. 10:e1004582). B. Nuclear localization of CRC1. crc1 fused with egfp and expressed from plasmid pSMAC_02795_EGFP. CRC1 co-localizes with the DAPI-labelled nuclei. Growth 2d on slides with BMM, scale bar 10 μm. (PDF 3836 kb) [file 12863_2018_702_MOESM7_ESM.pdf]

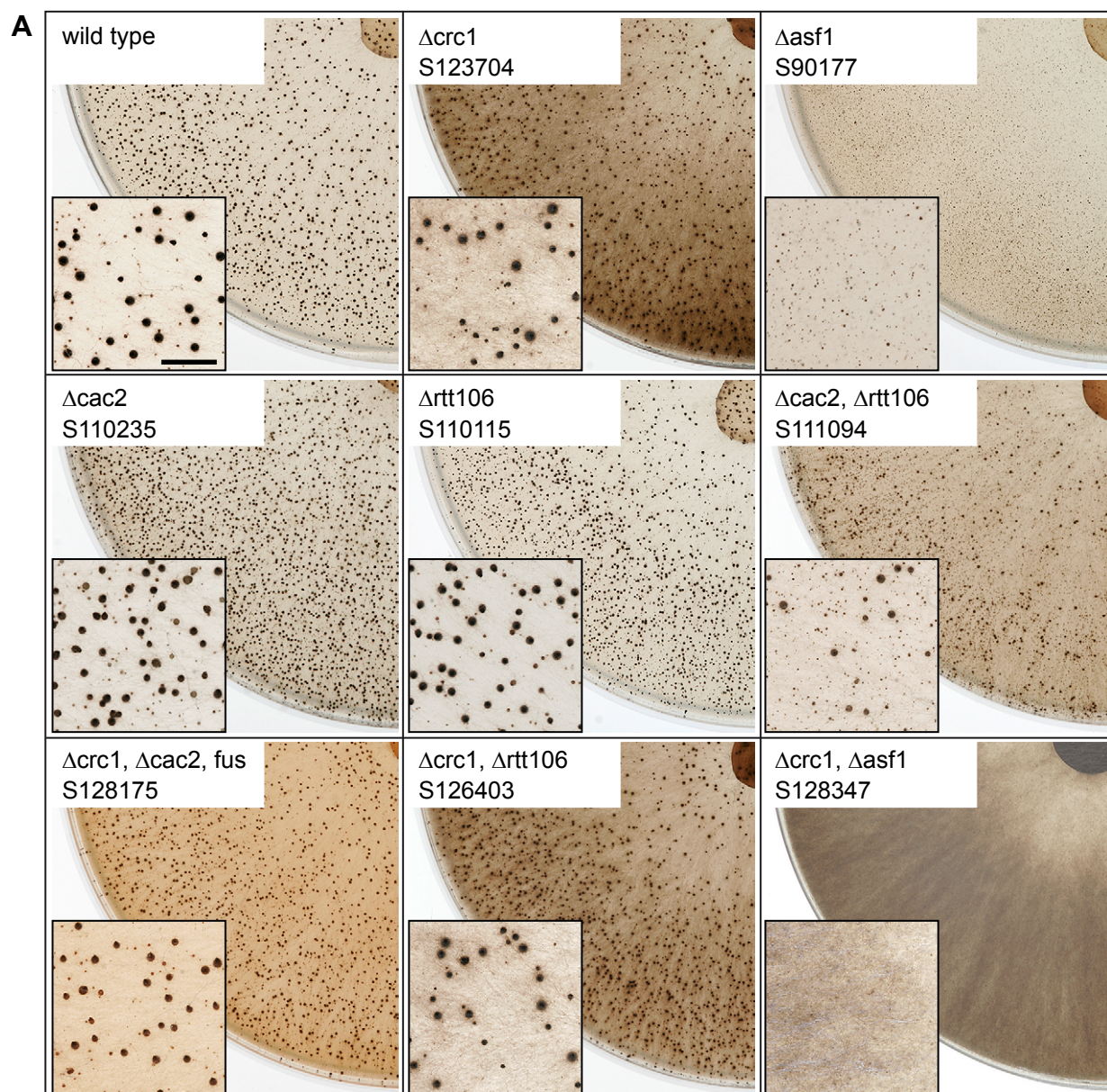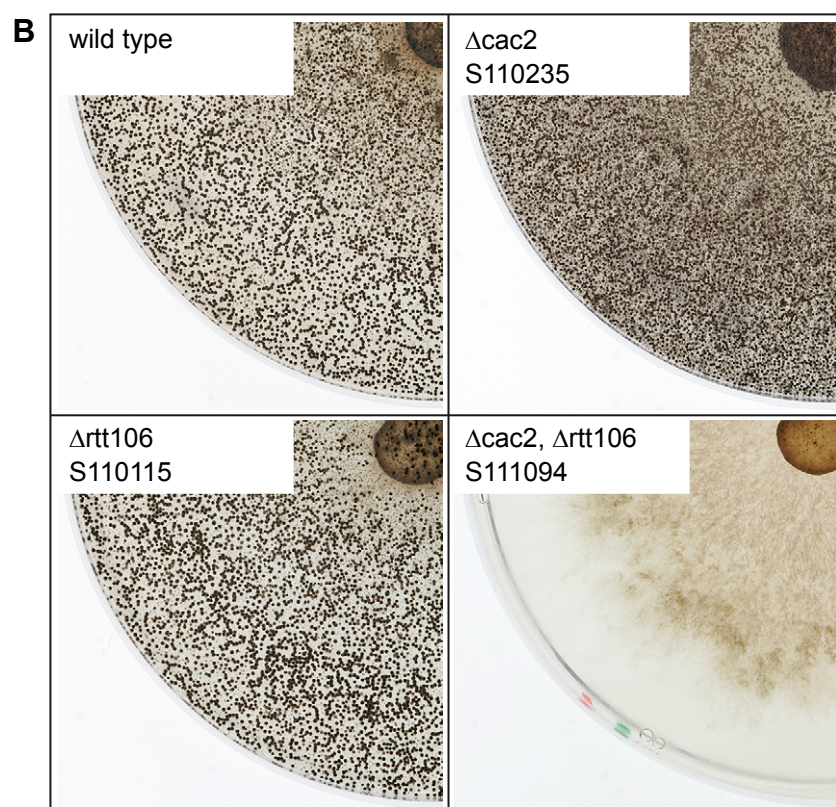

**Figure S8**

Supplement: Supplementary file 8 — Figure S8. Fruiting body development in chromatin modifier mutants. A. Strains were grown for 7 d on BMM at 25 °C. Photographs show fruiting body development on petri dishes, small boxes in lower left of each picture show enlarged sections of each overview. Scale bar in small boxes is 2 mm. B. Strains were grown for 7 d on SWG at 25 °C. Photographs show fruiting body development on petri dishes. (PDF 5774 kb) [file 12863_2018_702_MOESM8_ESM.pdf]

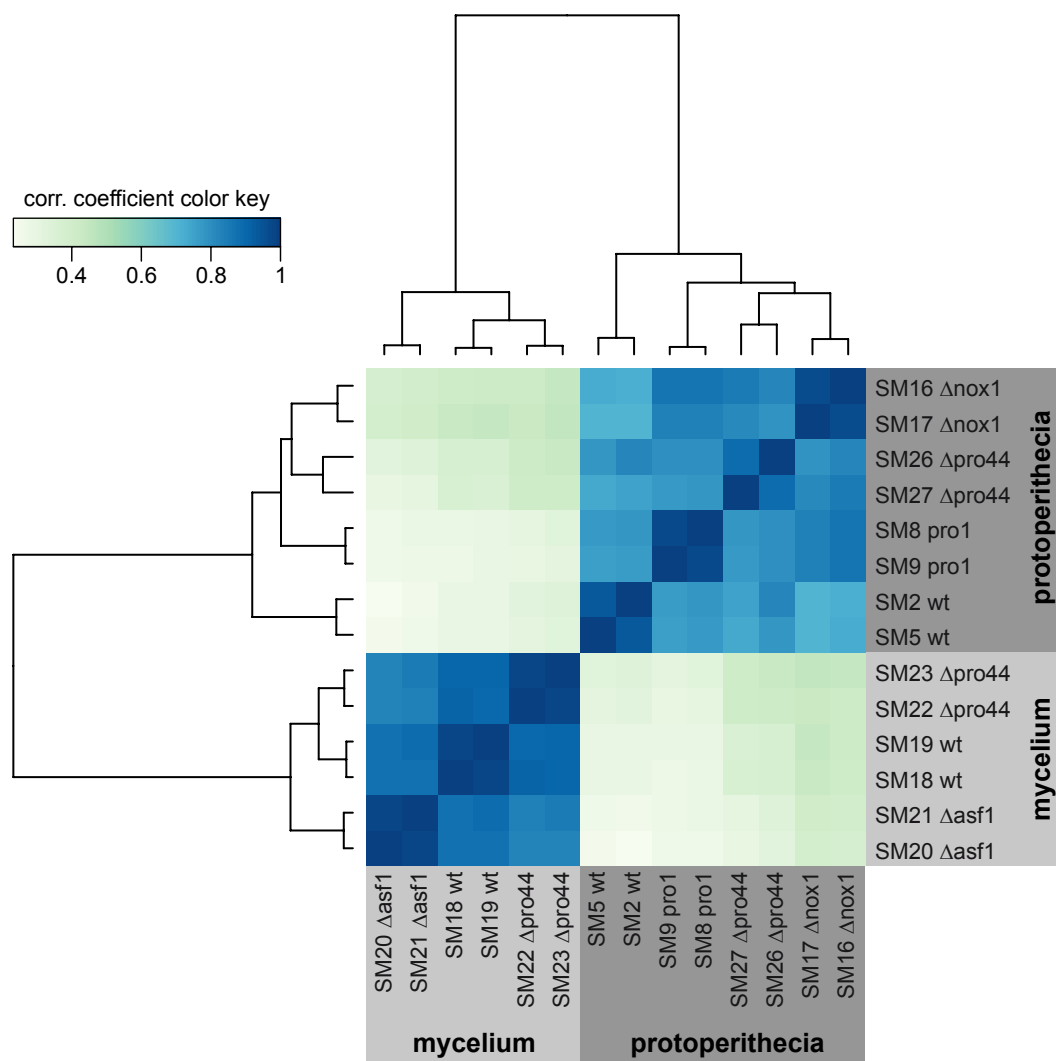

Figure S9

Supplement: Supplementary file 10 — Figure S9. Heatmap of Spearman‘s correlation coefficients for pairwise comparisons of the sets of RPKM values (log2 transformed) for each analyzed RNA-seq sample (two independent biological replicates per strain/condition). Included were RPKM values for all genes that had a measurement in all samples. Clustering and heatmap generation were done in R. (PDF 161 kb) [file 12863_2018_702_MOESM10_ESM.pdf]

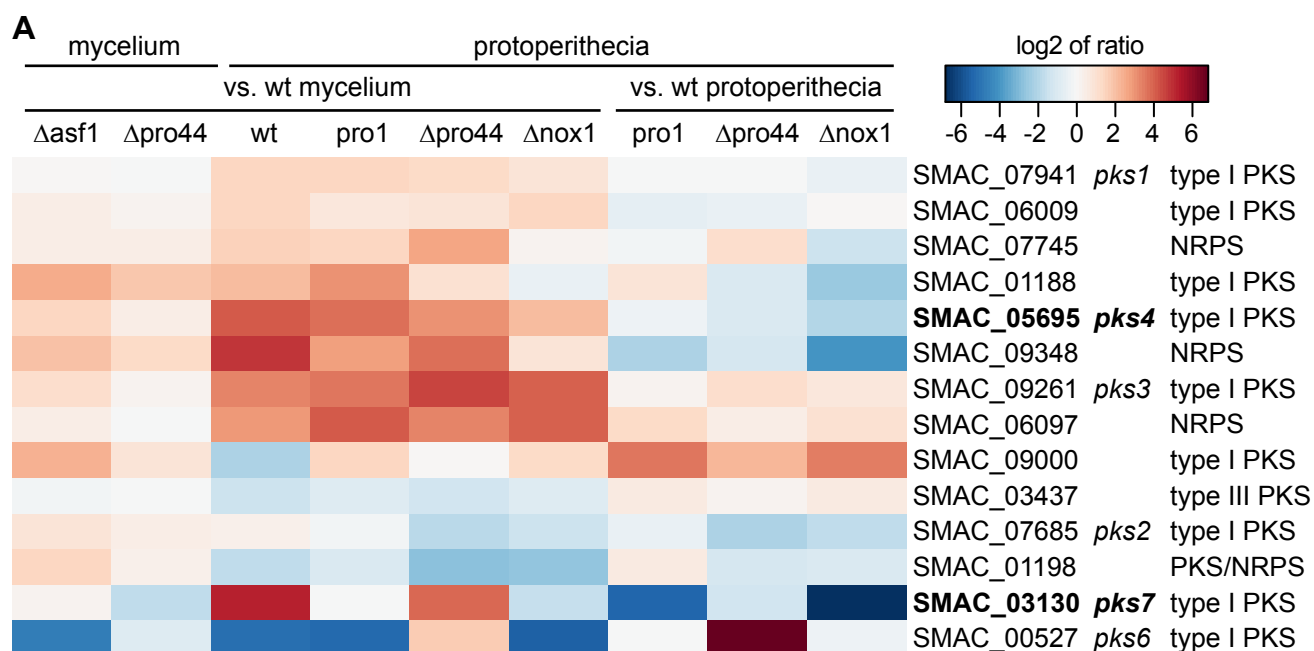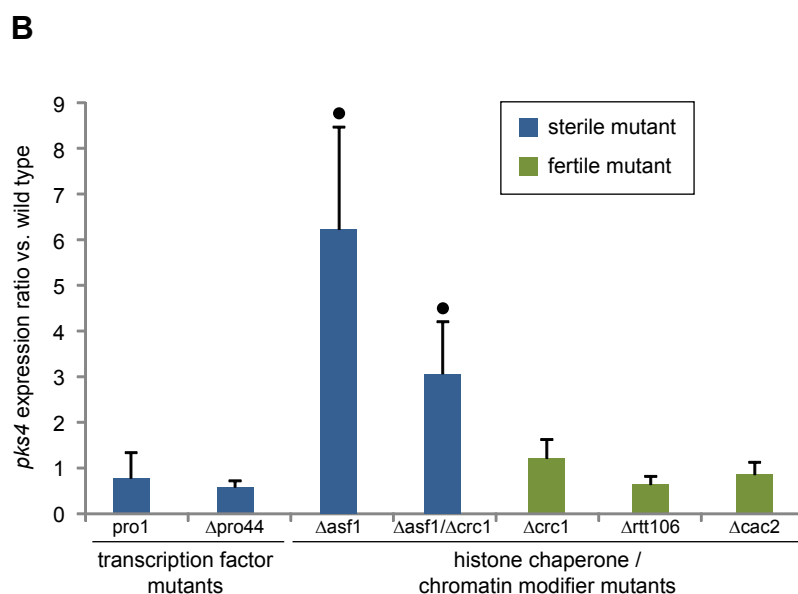

**Figure S10**

Supplement: Supplementary file 11 — Figure S10. Expression of pks and nrps genes in different mutants and tissues. A. Heatmap was generated based on the log2 ratios from the DESeq2 analysis of RNA-seq data. Two pks genes that were previously shown to be involved in fruiting body formation (pks4 for fruiting body formation and morphology, pks7 for melanin formation for black pigmentation of perithecia and ascospores) are given in bold. The results show that pks4 is indeed upregulated in Δasf1, but not Δpro44 mycelium. Furthermore it is upregulated in all protoperithecia samples compared to wild type, again showing that expression is not strongly dependent on pro44, pro1, or nox1. Overall, several pks and nrps genes are upregulated in Δasf1 mycelia, more than in Δpro44 mycelia, and about half of the genes are strongly upregulated in wild type protoperithecia. B. Expression of pks4 in transcription factor or chromatin modifier mutants. Quantitative RT-PCR analysis of pks4 expression was performed, ratios and standard errors (given as error bars) were calculated with REST. Dots indicate significantly differential expression (REST, p < =0.05). Data for pro1 and pro44 are from Schindler and Nowrousian 2014 (Fungal Genet Biol 68: 48–59). (PDF 199 kb) [file 12863_2018_702_MOESM11_ESM.pdf]

**A**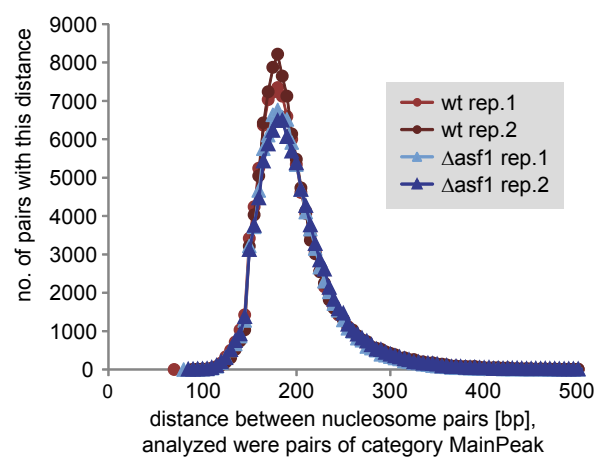**B**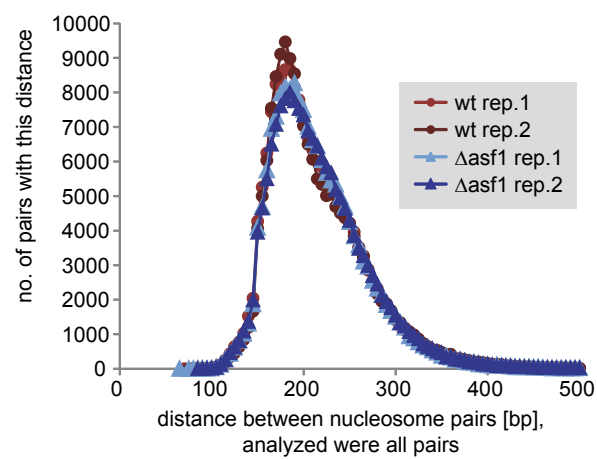**Figure S11**

Supplement: Supplementary file 12 — Figure S11. Distances between nucleosome pairs as determined by iNPS analysis of MNase-seq reads of wild type and Δasf1. Results are shown for two independent biological replicates for each strain. A. Analyzed were distances between pairs of nucleosomes of the iNPS category “MainPeak”. B. Analyzed were all nucleosome pair distances, irrespective of nucleosome type determined by iNPS (nucleosome types MainPeak, MainPeak:doublet, MainPeak+Shoulder, Shoulder). (PDF 157 kb) [file 12863_2018_702_MOESM12_ESM.pdf]

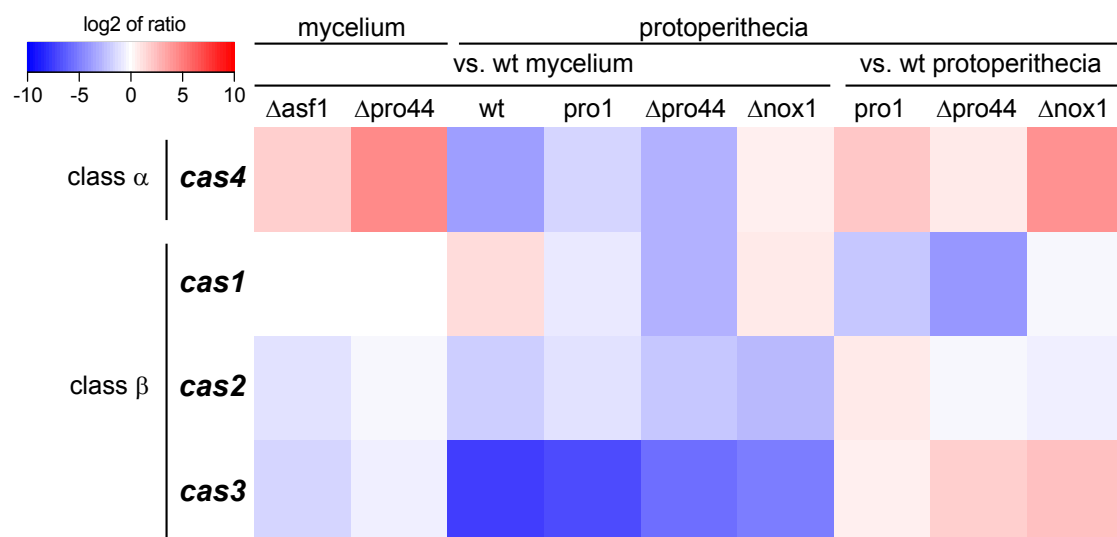

Figure S12

Supplement: Supplementary file 14 — Figure S12. Expression of carbonic anhydrase genes of S. macrospora in different mutants/conditions. Hierarchical clustering and heatmap generation of the log2 of fold ratios as determined in the DESeq2 analysis were done in R. (PDF 162 kb) [file 12863_2018_702_MOESM14_ESM.pdf]

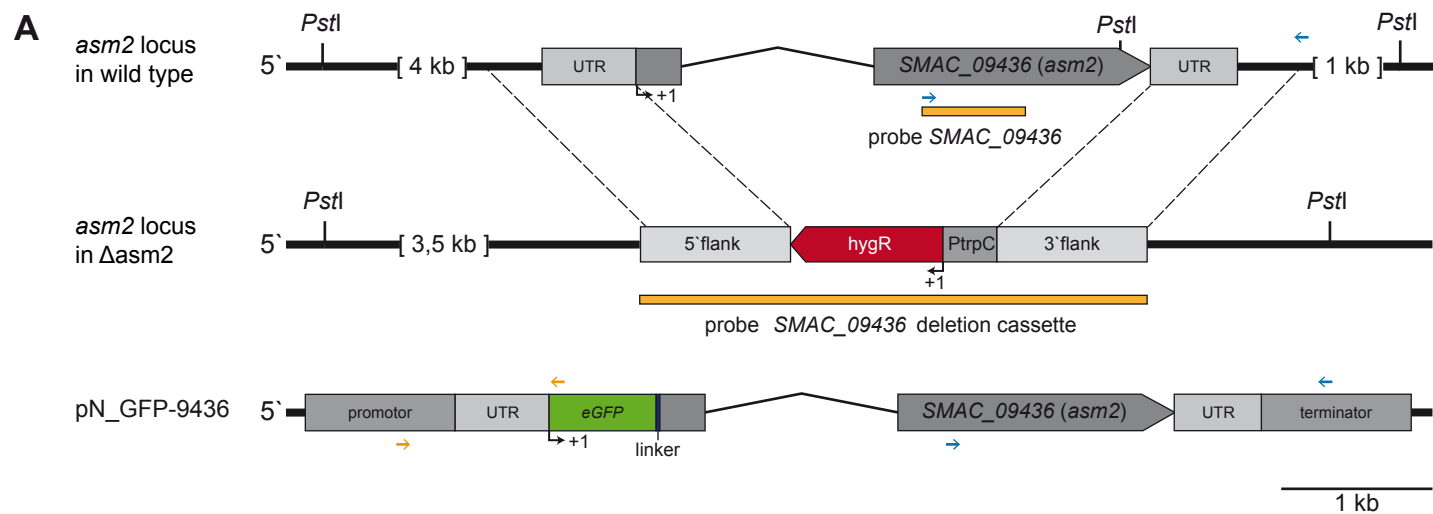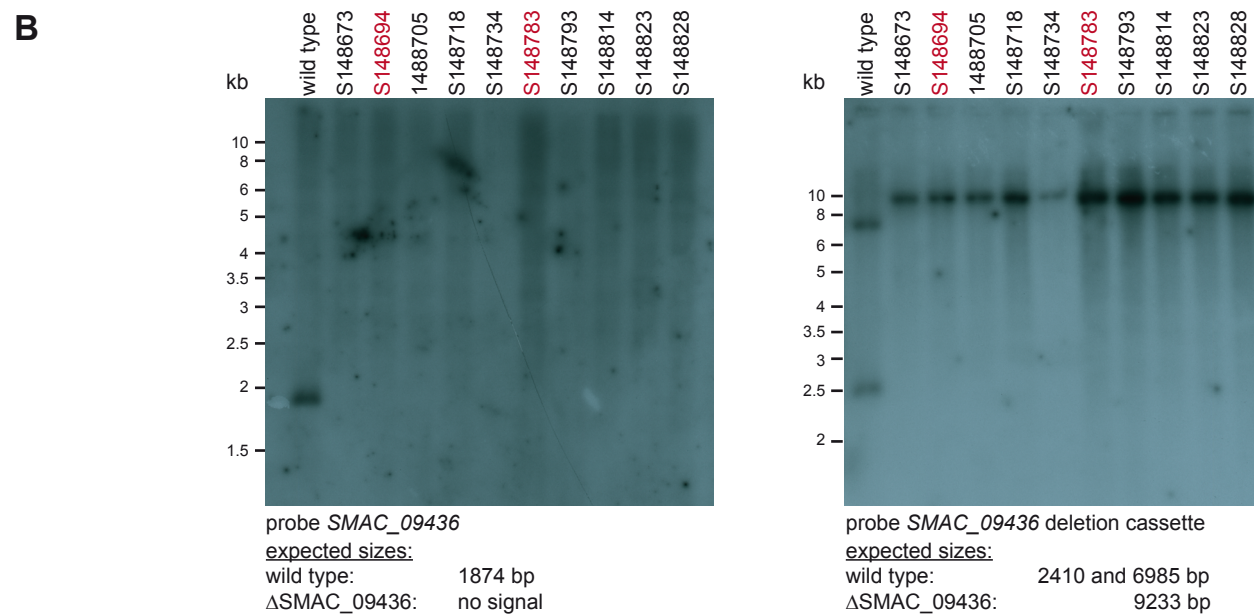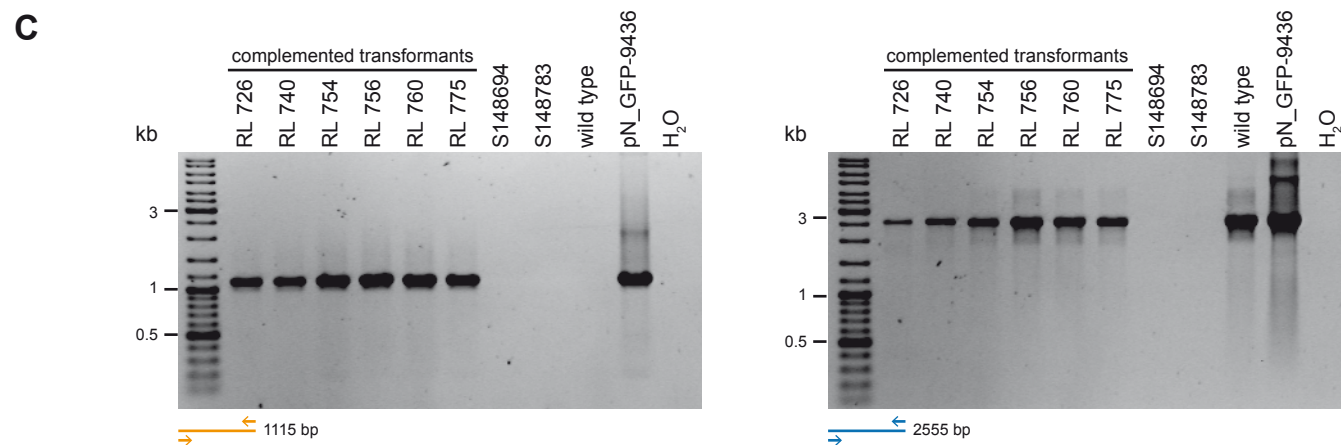

**Figure S14**

Supplement: Supplementary file 16 — Figure S14. Southern blot/PCR analysis of Δasm2 (ΔSMAC_09436) strains and complemented transformants. A. Overview of the asm2 genomic locus in the wild type and the corresponding deletion mutant, with the probes for the Southern blot indicated. Below, the SMAC_09436-containing region of complementation vector pN_GFP-9436 is shown. Blue and yellow arrows indicate primers for PCRs described in C. B. Southern blot analysis of the wild type and ten single spore isolates after digestion of genomic DNA with PstI. The blots were probed with the indicated probes. The resulting signals are as expected for both probes (sizes indicated below the blots). Deletion strains that were used in further experiments are labelled in red. C. Ectopic integration of complementation plasmid pN_GFP-9436 was confirmed by PCR. Six different single spore isolates from two different complemented transformants were analyzed with two different primer sets (see A). Spore isolates RL726 and RL740 are based on deletion strain S148783 as recipient for transformation, spore isolates RL754, RL756, RL760, and RL775 are based on S148694. As expected, with the primers amplifying a fragment from egfp to the asm2 promoter region (yellow arrows in A), amplicons were obtained only with the complemented transformants and the plasmid control. With primers amplifying a fragment from the C-terminal region to the terminator region of asm2, only complemented transformants, the wild type, and the plasmid control gave amplicons as expected. (PDF 1498 kb) [file 12863_2018_702_MOESM16_ESM.pdf]

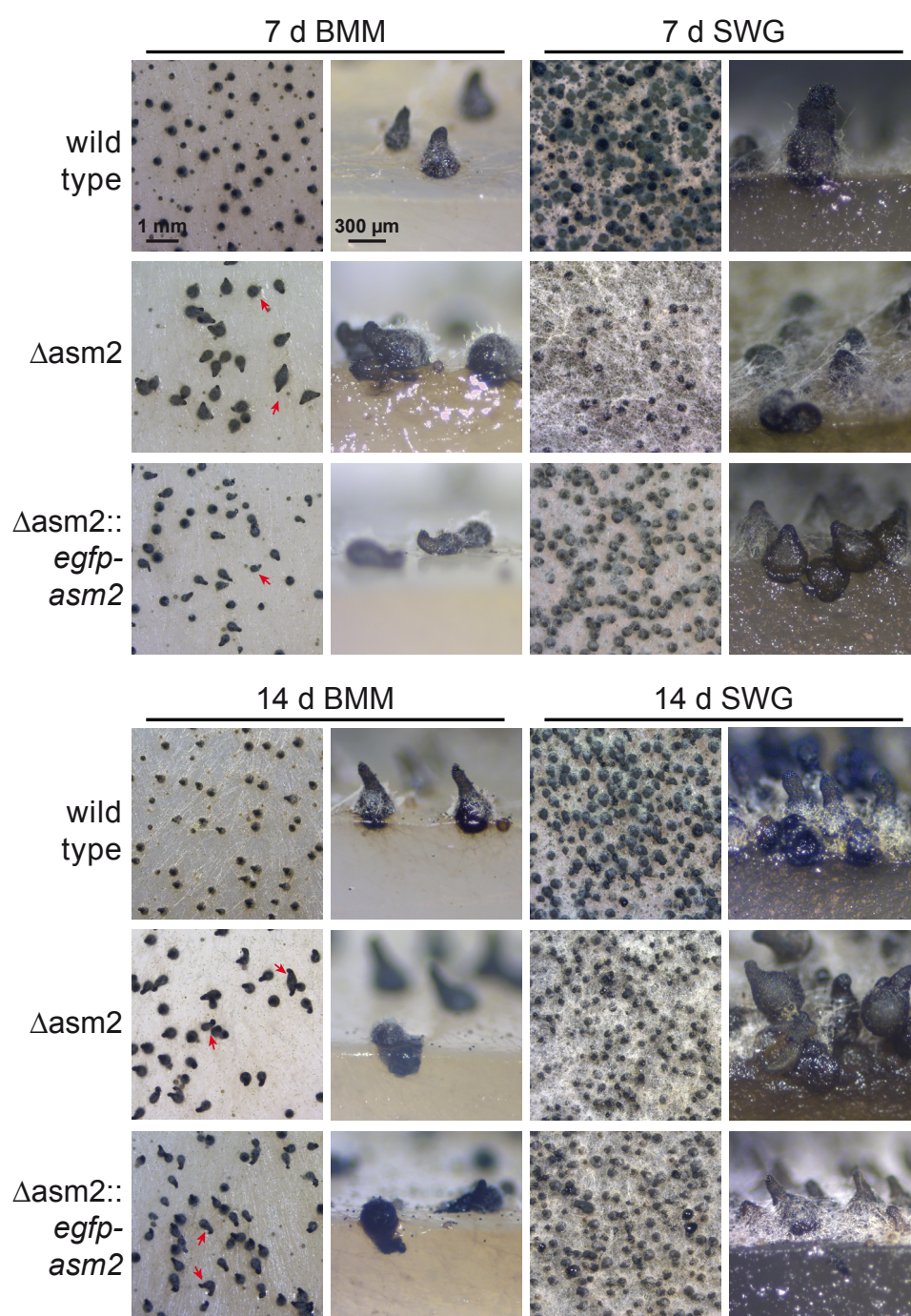

**Figure S15**

Supplement: Supplementary file 17 — Figure S15. Morphological characterization of Δasm2 (ΔSMAC_09436, spore isolate S148783) and a complemented transformant (spore isolate RL726). Strains were grown on full medium (BMM) or defined medium (SWG) at 25 °C for the indicated times. Scale bars are the same for all overview (left column for each time point, top view of perithecia growing on the surface of the agar medium) and detail (right column for each time point, side view of perithecia growing on the surface of the agar medium) pictures. In the asm2 deletion strain as well as the complemented transformant, perithecia are sometimes not arranged perpendicular to the growth surface as in the wild type (red arrows). (PDF 5730 kb) [file 12863_2018_702_MOESM17_ESM.pdf]
