# Supplementary material for: The transcription factor PRO44 and the histone chaperone ASF1 regulate distinct aspects of multicellular development in the filamentous fungus Sordaria macrospora
Source: BMC Genet. 2018 Dec 13;19:112. doi: 10.1186/s12863-018-0702-z (PMC6293562; doi:10.1186/s12863-018-0702-z)
Supplement: Supplementary file 5 — Figure S5. Southern blot analysis of Δcrc1 double mutants. A. Overview of the genomic locus in the different deletion mutants, with the probe for the Southern blot indicated. b. Southern blot analysis of the wild type and the Δcrc1, Δrtt106 double mutant S126403 after digestion of genomic DNA with HindIII. The blot was probed with the hph cassette. The resulting signals are as expected for the double mutant (6.8 kb band for the Δcrc1 locus and 3.7 kb band for the Δrtt106 locus) and the wild type (no signal). C. Southern blot analysis of the wild type and single ascospore isolates from crosses of Δcrc1 and Δasf1, or Δcrc1 and Δcac2, respectively. Genomic DNA was digested with HindIII. The blot was probed with the hph cassette. Double mutants Δcrc1, Δasf1 can be identified by bands at 6.8 kb for the Δcrc1 deletion and 3.5 kb band for the Δasf1 deletion. Double mutants Δcrc1, Δcac2 can be identified by bands at 6.8 kb for the Δcrc1 deletion and 4.2 kb band for the Δcac2 deletion. Double deletion strains that were used in further experiments are labelled in red. (PDF 1061 kb) [file 12863_2018_702_MOESM5_ESM.pdf]

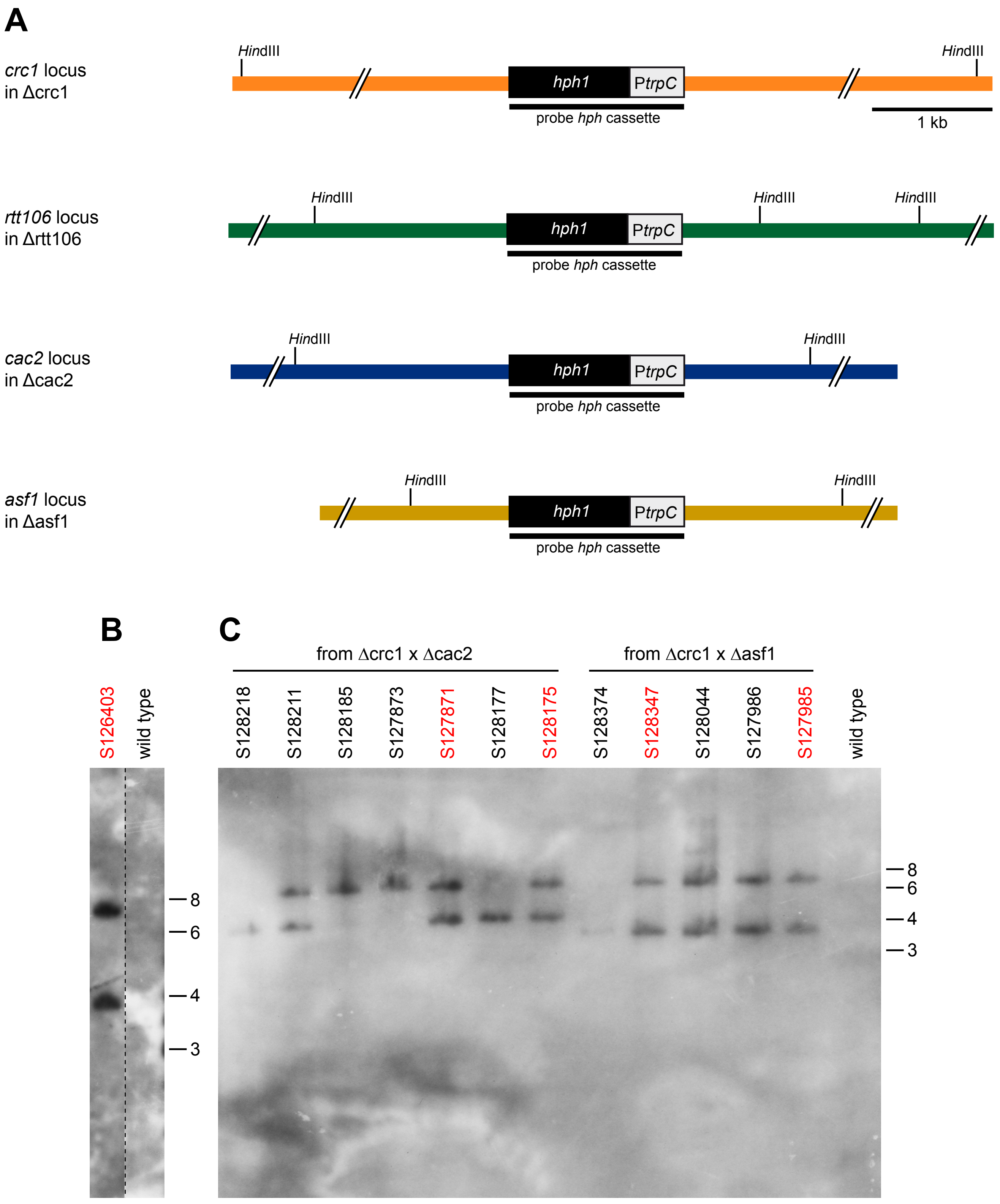

*Hind*III digest, probe: *hph* cassette

Figure S5
