# Supplementary material for: The transcription factor PRO44 and the histone chaperone ASF1 regulate distinct aspects of multicellular development in the filamentous fungus Sordaria macrospora
Source: BMC Genet. 2018 Dec 13;19:112. doi: 10.1186/s12863-018-0702-z (PMC6293562; doi:10.1186/s12863-018-0702-z)
Supplement: Supplementary file 15 — Figure S13. Multiple alignment of SMAC_09436 (ASM2) orthologs. Orthologs were determined by bidirectional BLASTP analyses. Proteins from the Sordariomycetes Sordaria macrospora (S.m., SMAC_09436), Neurospora crassa (N.c., NCU010258), Podospora anserina (P.a., CDP26737.1), Magnaporthe oryzae (M.o., XP_003720415.1), Fusarium graminearum (F.g., FGRAMPH1_01T14721), and Trichoderma reesei (T.r., XP_006961730.1) were aligned with ClustalX. No clear orthologs outside of the Sordariomycetes could be identified. The GAL4 (GAL4-like Zn2Cys6 binuclear cluster DNA-binding) domain and the fungal_TF_MHR (fungal transcription factor regulatory middle homology region) domain in SMAC_09436 are indicated by black and grey bars, respectively, above the sequence. (PDF 176 kb) [file 12863_2018_702_MOESM15_ESM.pdf]

```
S.m.    1 MSS-VVRSTALRAGGACVRCRKGKTKCVYENGRAPCKNCAKGMHECYLPSESMAHAHGGVAPSNR--QQRPRESLPSERIVPT 80
N.c.    1 MSS-VVRSTALRAGGACVRCRKGKTKCVYENGRAPCKNCAKGMHECYLPSESMAHAHGGVAPSNR--QQRPRESLPNERIVPT 80
P.a.    1 MAS-VVRSTALRAGGACVRCRKGKTKCVYENGRAPCKNCAKGMHECYLPSESMAHGGHGVSPARM--QHRIRESLPSERVVSS 80
M.o.    1 MAS-VVRATALRAGGACVRCRKGKTKCVYENGRAPCKNCAKGMHDCYLPSESMSHGGHGVSPARV--PQRSRESLPSDARSVG 80
F.g.    1 MASSIRSTTALRAGSACTRCRKGKTKCVYESGRPPCKNCAKGMHDCYLPSESLQHHGGGSPARHANHPHRPRDNVPAAAAPAA 83
T.r.    1 MSSGVVRSTALRAGGACVRCRKGKTKCVYENGRAPCKNCAKGMHECYLPSESMAHHHGHSPARHT-AAHRPRDALPASGPGAS 82

S.m.   81 SAGDRGGP--PSVGASRHVSTTNEKLTPELLQECERVINKTLPACVAFHKPSCLQQLKNASMEWTLVNALLTTAARHSPAMIR 161
N.c.   81 SAGDRGGP--PSVGASRHVSTTNEKLTPELLQECERVINKTLPACVAFHKPSCLQQLKNASMEWTLVNALLTTAARHSPAMIR 161
P.a.   81 SAVDRQVPAGPSSSVSRHASAAHEKLTPELMTECERVISKTLPACVAFHKPTFLVALKNASMEPTMVNALLTTAARHSPAMIR 163
M.o.   81 PSAERNVGGQGGSSASRHTGTTSEKLTPELLAECERVVSKTIPACVAFHKPSFIQQLKGNTMDLTMVNALLTMASRHSPVLIR 163
F.g.   84 E-RQPVVG----AAPTRHAQTGSDKLTPELISECERVVSKTFPACVAFHKPSFVQQLKSASLDAALVYGLLTCAARSSPSLIR 161
T.r.   83 DARQPVVG----SGGARHAQATSEKLTPELIAECERVVSKTFPACVAFHKPSFVQQLKNASLESSLVYGLLTCAARSSPTLIR 161

S.m.  162 RYGGQLGGAGAAEHFAQKAGQLVMQSLASPSLADIQALCLLVIHEWGCREAVRAYIYLGQAARMIQMYRIVSAQQRNSDPDQF 244
N.c.  162 RYGGQLGGAGAAEHFAQKAGQLVMQSLASPSLADIQALCLLVIHEWGCREAVRAYIYLGQAARMIQMYRIVSAQQRNSDPDQF 244
P.a.  164 RYGGHGSSSTAAEHFASKTINLVMQNLDTPSLADIQSVCLLILHEWGCRNAVRAYTYLGLAARMAQMYRLVHHHNSTNEPDQF 246
M.o.  164 RYGSHGGATGAAEHFALKSINGIMQGLDHPSLADIQAICLLVIHEWGSRNAVRAYIYLGQAARMAQMYRVAHAHEQ-SEPQQF 245
F.g.  162 RYGG---PTQAAETFAAKAMTLINQNLDHPNLVDIQALCLIIIHEWGSRNAVRAYIYLGQAARMVQMYRILNSHHPSPDADLF 241
T.r.  162 RYGGN--PTAAAETFAAKAISLINSNLDQPSLADVQALCLVIIHEWGSRNAVRAYVYLGQAARLLQMYRIINSHHAPDQTDPF 242

S.m.  245 LQDESFRRTLWLIYILDCFLTSSPGRRPALSTHDVVDIALPCADMNFNFGSPVVVRTIGGAAPS----HAEPGAALSEVGEFG 323
N.c.  245 LQDESFRRTLWLIYILDCFLTSSPGRRPALSTHDVVDVALPCADMNFNFGSPVVVRTISGAAPS----HAEPGAALSEVGEFG 323
P.a.  247 QMEESFRRTLWLIYILDCFLTSSPGRHPALTHLDVQDVSLPCLDMNYNFTSPVHVRTLSGAPPAG---LKDSSAQLSEVGEFG 326
M.o.  246 VQSESFRRTMWLIYILDCFLTASPGRHPALSQRDVRDMALPCLDMNYTFDSPAFVRTLDGTLPS----QAPHGTVPAEVGEFG 324
F.g.  242 LRDESLRRTVWLIYILDCLLTSTPGRYSALSPHDTADVSLPCSDINFAFGNAVFVKTLRQQLDPN---AVPPGQPPSEIGEFG 321
T.r.  243 LRDESFRRTLWLIYILDCLLTCTPGRYPALAIQDTAEVALPCSDINFAFGNTVYVKTLHQQLAQQNNHSSPSHVPSGEVGEFG 325

S.m.  324 HIVLATRAWRNVVEMMTTTTLETFSDTQCLALEADIDTLRQTLPPHFADKPGQINLHITMGSGYTYAMIHCLLNCGTIFVNRR 406
N.c.  324 HIVLATQAWRNVVEMMTTTTLETFDDQRCLGLEADIDTLRQSLPPHFADKPGQINLHITMGSGYTYAMIHCLLNCGTIFVNRR 406
P.a.  327 HIVLATKAWRQVVEMMTTTTLATFSDERCVQLEQDIEVLRQSLPMHFADKPNNINLHITMGSGYTYAFIHCLLNCGTIFVNRR 409
M.o.  325 HFVLATKAWRNVIELMTTTTLATFTEDRCQILEAEIENIRSSLPPHFSDKPGQIAVHITMGSGYTYAMIHCLLHCASIFLNRR 407
F.g.  322 YIVLASTIWRDVVAMLTTTTLASFREEDCSDLIAKIEGLRATLPMQFVDKPGQINLHMTMGSGYTFAMLHCLLHCATVFVHRR 404
T.r.  326 YIVLAATIWRDVVGTLG---ISPFREEDCTDLVIKIERLKASLPMQFVDKPGQINLHMTMGSGYTYAMLHCLIHCATIFVHRR 405

S.m.  407 RILQVVTDENFTIDAWRMASHTHMQTVDRIFAASHSIIHSLLALEHGADKDIMVCFPIFMLFSAFTAGSTVAYLTLKSLAPST 489
N.c.  407 RILQVVTDENFTIDAWRMASHTHMQTVDRIFAASHSIIHSLLALEHGADKDIMVCFPIFMLFSAFTAGSTVAYLTLKGLAPST 489
P.a.  410 RMLQVVTDENFSIDLWRGSSHTHVQTVDKIFAASHSIIHSLLALETGADKDSILCFPLFMLFAAFTAGSTVAYLTLKGLAPAN 492
M.o.  408 RILQDVSAEGFSADAWRAS--GRAQLADQIFGAAHSIVSMLTALEGGAEKDSILCFPIFMLFAAFTSGAAVAYLQLKGLTPAD 488
F.g.  405 RLLQEVTSSNFNLESFRLN-ARCHDIIDRLFTSCHGTISLLTAVEAGSEKDHSPCFPIFMLFSAFTASATVAYLSLKGLTPPN 486
T.r.  406 RLLEDVTSPSFNLEAYRMT-SRCHDIIDRLLTSCHGTLTLLTAIESGAEKDHATCFPIFMLFSAFTASATVAYLSLKRLTPPN 487

S.m.  490 VTESASSIVRDSLRLCQDGSESWPLVTPWARHLTVMSKVLRDMKVLGRDREAREDSRATLHAASPHIKDDVATPPDNTNHPEA 572
N.c.  490 VTESASSIVRDSLRLCQDGSESWPLVTPWARHLTVMSKVLRDMKVLGRDREAREDSRATMHATSPHIKDDMATPPDNTN-PEA 571
P.a.  493 SSESAFNIVRDSVRMCQDGAESWPLVIPWQRHLSVMSKVLRDVKSAPRDK-AREDSKK--RALSPSVKDDNVS-QTDTN-PDA 570
M.o.  489 VRETTSTIVRDVFRFLKDGADCWPLIVPWQRHLSVMAKVLKEYTARIDREESPDGAKAR---ASPSIKDDVSSQPDTST-ADV 567
F.g.  487 AVETAAHIVKDGLRFMSDGTENWPLMGSWLRHLTVMQRVLNNDAAAANGGSSLRHSSAS--HGAGGAKDEISSNADTNP--DV 565
T.r.  488 AVETAAHIVKDGLRFMRDGNESWPLLNSWLRHLTVMQRVLN------SSQGSPHEAHPH--MSSAGIKDEMSSNADTNP--DA 560

S.m.  573 MEFDTVQPNSTGLGSVGPAAASPGAPTPTIEGAGGSEVGETPLVRRPQGITTINGGSVGAVDTRVATTSPPPHATPVLQHQQM 655
N.c.  572 MEFDTVPPNSTAPGSQPPNAASPGLPTPTIEGAGGSEVGETPLVRRP-GITTINGGSVGAVDTRVATTSPPPHATPVLQHQQM 653
P.a.  571 MDYEQPDVSAAGP-----------QAVPTTEGRASE-----PPVPRKTGITTINGG---PVAVSTPADSPPPPVP-----VVA 629
M.o.  568 MDYDQTDNSTGHG-----SAAHGGQPHPTIEGGRDG--SEPPAAPRRPGIATINGGSAGNSTPATGSPAPMPARES----GSM 639
F.g.  566 MDYDQTNRAGSVS--------GQGPN-RSLSESVRGDSEPPVVLARRPGIATINGG---SGGVSTPTTSPPPSNTP---HGAS 633
T.r.  561 MEYDQPAASVAPS--------QQGVEHRSVSGSARGESEPPITLPRRPGVTTING----NNIATPETVSPPPPGAP---QANM 628

S.m.  656 KVDSPSAQSSVSNQGGANAQGASVPPSGADAVDMTANELCQAFERQLLELDDLAAFMGGGV-----                   716
N.c.  654 KVDSPSAQSSVSNQGVANAQGASVPPGGADAVDMTANELCQAFERQLLELDDLAAFMGGGV-----                   714
P.a.  630 KVDSPSPASPGSALG--GVQSSSTGP-DVPDVDMTALELCAAFERQLLELDDLAAFMGGGVGNAPS                   692
M.o.  640 KIDSPDPNDAASGGSSSTAKNGSNGPPAQQEPAIDSADLCAAFERQLLELDDLAAFMGGGV-----                   700
F.g.  634 GMSGIKQTSPEMANGIVPPQDG-----QTTSQDMTAPELCQAFERQLLDLDDLAAFMGGGV-----                   689
T.r.  629 QVHDVKQQSPEDAQNGSPHDGG-----QTTSQDMTSPELCLAFERQLMDHDDLAAFMGGGV-----                   684
```

Figure S13
