# Supplementary material for: The transcription factor PRO44 and the histone chaperone ASF1 regulate distinct aspects of multicellular development in the filamentous fungus Sordaria macrospora
Source: BMC Genet. 2018 Dec 13;19:112. doi: 10.1186/s12863-018-0702-z (PMC6293562; doi:10.1186/s12863-018-0702-z)
Supplement: Supplementary file 18 — Table S3. Sordaria macrospora strains used in this study. (PDF 288 kb) [file 12863_2018_702_MOESM18_ESM.pdf]

Table S3. *Sordaria macrospora* strains used in this study

Strain	Relevant genotype and phenotype	Reference or source[a]
wild type	wild type	Culture collection
fus	spore color mutant	Nowrousian et al. (2012)
S96888	Δku70	Pöggeler and Kück (2006)
S90177	Δasf1; sterile	Gesing et al. (2012)
S110115	Δrtt106; fertile	Gesing et al. (2012)
S110235	Δcac2; fertile	Gesing et al. (2012)
S106095	Δpro44; sterile	this study
S108950	S106095 + pRSnat-pro44-NA; fertile	this study
SFA198	S106095 + pFA20; fertile; (Δpro44 + Ppro44::*pro44_egfp*)	this study
SFA4019	S106095 + pFA30; fertile; (Δpro44 + Pgpd::*pro44_ntap*)	this study
T8.1	wild type + pDS23 + pBH2B; fertile; (wt + Pgpd::*egfp*::Ttrpc; Pgpd::*hh2b_mKal*::Ttrpc)	this study
S135826	Δpro44 + Pgpd::*pro44_ntap*::nat + Ppro44::*pro44_egfp* (ascospore isolate from cross of S198 and S4019)	this study
S111081	Δrtt106, Δcac2, fus; fertile	this study
S111083	Δrtt106, Δcac2; fertile	this study
S111094	Δrtt106, Δcac2; fertile	this study
S123617	Δcdp1; fertile	this study
S123704	Δcdp1; fertile	this study
S126403	Δcdp1, Δrtt106; fertile	this study
S128347	Δcdp1, Δasf1; sterile	this study
S127985	Δcdp1, Δasf1, fus; sterile	this study
S127871	Δcdp1, Δcac2, fus; fertile	this study
S128175	Δcdp1, Δcac2, fus; fertile	this study
S122553	S111083 + pRTT106-EGFP + pRH2B; fertile	this study
S122560	S111083 + pCAC2-EGFP2 + pRH2B; fertile	this study
S148694	Δasm2; sterile	this study
S148783	Δasm2; sterile	this study
RL726	S148783 + pN_GFP-9436 (Δasm2 + Pasm2::*egfp*::*asm2*::Tasm2)	this study
RL740	S148783 + pN_GFP-9436 (Δasm2 + Pasm2::*egfp*::*asm2*::Tasm2)	this study
RL754	S148694 + pN_GFP-9436 (Δasm2 + Pasm2::*egfp*::*asm2*::Tasm2)	this study
RL756	S148694 + pN_GFP-9436 (Δasm2 + Pasm2::*egfp*::*asm2*::Tasm2)	this study
RL760	S148694 + pN_GFP-9436 (Δasm2 + Pasm2::*egfp*::*asm2*::Tasm2)	this study
RL775	S148694 + pN_GFP-9436 (Δasm2 + Pasm2::*egfp*::*asm2*::Tasm2)	this study

[a]Culture collection: Lehrstuhl für Allgemeine und Molekulare Botanik, Ruhr-Universität, Bochum, Germany

References:

Gesing S, Schindler D, Fränzel B, Wolters D, Nowrousian M (2012) The histone chaperone ASF1 is essential for sexual development in the filamentous fungus *Sordaria macrospora*. Mol Microbiol 84:748-765

Nowrousian M, Teichert I, Masloff S, Kück U. 2012. Whole-genome sequencing of *Sordaria macrospora* mutants identifies developmental genes. G3 (Bethesda) 2:261-270

Pöggeler S, Kück U. 2006. Highly efficient generation of signal transduction knockout mutants using a fungal strain deficient in the mammalian *ku70* ortholog. Gene 378:1-10
