# Supplementary material for: The transcription factor PRO44 and the histone chaperone ASF1 regulate distinct aspects of multicellular development in the filamentous fungus Sordaria macrospora
Source: BMC Genet. 2018 Dec 13;19:112. doi: 10.1186/s12863-018-0702-z (PMC6293562; doi:10.1186/s12863-018-0702-z)
Supplement: Supplementary file 19 — Table S4. Oligonucleotides used in this study. (PDF 318 kb) [file 12863_2018_702_MOESM19_ESM.pdf]

Table S4. Oligonucleotides used in this study.

Name	Sequence 5'-3'	Remarks
SMAC_02795-ko1	GTAACGCCAGGGTTTTCCCAGTCACGACGaagcttGGTAGCCTGGAACATGGGCTGCAAC	amplification of upstream region of *cdp1* for cloning of deletion vector
SMAC_02795-ko2	cgagggcaaaggaatagggttccgttgaggGAGTGCAGGCGACGTTAACCACGTC	amplification of upstream region of *cdp1* for cloning of deletion vector
SMAC_02795-ko3	gcccaaaaatgctccttcaatatcagttgcACGAAGATGGCGCTTTGCTAGGTGC	amplification of downstream region of *cdp1* for cloning of deletion vector
SMAC_02795-ko4	GCGGATAACAATTTCACACAGGAAACAGCaagcttGTTGATACGACAAAACAAGTGTACATCC	amplification of downstream region of *cdp1* for cloning of deletion vector
SMAC_02795-ver1	cgcactctctccacttctgtgtcc	for verification of *cdp1* deletion
SMAC_02795-ver2	GGAAAATAATACGAGCCAAGTAGC	for verification of *cdp1* deletion
SMAC_02795-ver3	actcggctgcatggtgagcttgg	amplification of probe for Southern blot analysis of *cdp1* ORF
SMAC_02795-ver4	gtggtgataagcagcctagaaagc	amplification of probe for Southern blot analysis of *cdp1* ORF
SMAC_02795_ORF1	CGCAGCTTGACTAACAGCTACAGATCATGTACGCAGCACAGCCAAACGGCGTTG	amplification of first part of *cdp1* ORF for cloning of *egfp* fusion construct
SMAC_02795_ORF2	GAACAGCTCCTCGCCCTTGCTCACCATTTGTGGTTGATTGGACCACCACGTAGCAG	amplification of first part of *cdp1* ORF for cloning of *egfp* fusion construct
SMAC_02795_ORF3	GACCACACAACCGACCAGCTTGG	amplification of second part of *cdp1* ORF for cloning of *egfp* fusion construct
SMAC_02795_ORF4	TATTCACGATTTCCGGTAAGCTGC	amplification of second part of *cdp1* ORF for cloning of *egfp* fusion construct
5´09436_fw	GCCAGGGTTTTCCCAGTCACGACGGAATTCccttttggtccagtccggca	amplification of upstream region of *asm2* for cloning of deletion vector
5´09436_bw	GGAATAGGGTTCCGTTGAGGttcggtgaccaaacgggctg	amplification of upstream region of *asm2* for cloning of deletion vector
3´09436-hph_fw	ATGCTCCTTCAATATCAGTTgccggtgtccatgacgcggg	amplification of downstream region of *asm2* for cloning of deletion vector
3´09436-bw	ATTTCACACAGGAAACAGCGAATTCgaacgacaggtacttaccgt	amplification of downstream region of *asm2* for cloning of deletion vector
hph-5´09436_fw	cagcccgtttggtcaccgaaCCTCAACGGAACCCTATTCC	amplification of *hph* cassette for cloning of *asm2* deletion vector
hph-3´09436_bw	cccgcgtcatggacaccggcAACTGATATTGAAGGAGCAT	amplification of *hph* cassette for cloning of *asm2* deletion vector
9436_5`_fw	gcaaaacgcccagcaccatc	for verification of *asm2* deletion
9436_3`_rv	tatgggaggactcgttcgtaggtg	for verification of *asm2* deletion
HR-P9436_fw	cgaggtcgacggtatcgataagcttacaaatgacaagccccgtccgctgt	amplification of upstream region of *asm2* for cloning of complementation vector
HR-P9436-gfp_rv	acagctcctcgcccttgctcaccatttcggtgaccaaacgggctggctgg	amplification of upstream region of *asm2* for cloning of complementation vector
HR-gfp-link9436_fw	gatctcaggtacctggaattcgagcatgtcgtcggtcgttaggtccacgg	amplification of *asm2* (coding regions and intron) and downstream region for cloning of complementation vector
HR-T9436_rv	ggagctccaccgcggtggcggccgcgttct	amplification of *asm2* (coding regions and

	taccccaagttactcatctt	intron) and downstream region for cloning of complementation vector
SMAC_01629_ORF1	CGCAGCTTGACTAACAGCTACAGATCatgaaagccactcccttgatcatcaactg	amplification of *cac2* ORF for cloning of *egfp* fusion construct
SMAC_01629_ORF2	GAACAGCTCCTCGCCCTTGCTCACCATcgccttgcgcagatcactactgctgg	amplification of *cac2* ORF for cloning of *egfp* fusion construct
SMAC_03589_ORF1	CGCAGCTTGACTAACAGCTACAGATCatggcagccaagctcgactcccagacg	amplification of *rtt106* ORF for cloning of *egfp* fusion construct
SMAC_03589_ORF2	GAACAGCTCCTCGCCCTTGCTCACCATttcttccaatccctcgcccatatcctcat	amplification of *rtt106* ORF for cloning of *egfp* fusion construct
3223_5fw_IT	GTAACGCCAGGGTTTTCCCAGTCACGACGGAATTCCCAAGGTACCTTACTGGACC	amplification of upstream region of *pro44* for cloning of deletion vector
3223_3rv_IT	CGAGGGCAAAGGAATAGGGTTCCGTTGAGGATGGCGTCGATGACCCAAAG	amplification of upstream region of *pro44* for cloning of deletion vector
3223_3fw_IT	GCCCAAAAATGCTCCTTCAATATCAGTTGCTCGACAAACTCGATTGCAGCC	amplification of downstream region of *pro44* for cloning of deletion vector
3223_3fv_IT	GCGGATAACAATTTCACACAGGAAACAGCGAATTCGGTTGATTTCCCACATGGAAGG	amplification of downstream region of *pro44* for cloning of deletion vector
NTAP_pro44_fw	TAAGCTTATCGATACCGTCGACTCCATGGAAGCGGGCGATTCTGCTGCTAGG	amplification of *pro44* for cloning of pFA30
NTAP_pro44_rv	CAAACCCACCGACGATCGACACTAGGATCCACTTAACGTTACTGAAATCA	amplification of *pro44* for cloning of pFA30
f1_P3223_fw	GTGCGGGCCTCTTCGCTATTACGCCAATACAGGTACACCTTTTCCCACTA	amplification of upstream regions of *pro44* for cloning of pFA20
egfp_P3223_rv	ACAGCTCCTCGCCCTTGCTCACCATAGCGGGTCTTAATAAAATAAATGGC	amplification of upstream regions of *pro44* for cloning of pFA20
P3223_egfp_fw	CATTTATTTTATTAAGACCCGCTATGGTGAGCAAGGGCGAGGAGCTGTTC	amplification of *egfp* for cloning of pFA20
Egfp_pro44_rv	TAGCAGCAGAATCGCCCGCTTCCATCTTGTACAGCTCGTCCATGCCGAGA	amplification of *egfp* for cloning of pFA20
pro44_egfp_fw	TCTCGGCATGGACGAGCTGTACAAGATGGAAGCGGGCGATTCTGCTGCTAGGCAAA	amplification of *pro44* for cloning of pFA20
pro44_T3223_rv	TGTATTTTTCTGCCGGATTATAAATCTAGTGTCGATCGTCGGTGGGTTTG	amplification of *pro44* for cloning of pFA20
T3223_fw	CAAACCCACCGACGATCGACACTAGATTTATAATCCGGCAGAAAAATACA	amplification of downstream regions of *pro44* for cloning of pFA20
T3223_rv	ATAACAATTTCACACAGGAAACAGCCTTTGCGCGCGGTATATAGAGTTAT	amplification of downstream regions of *pro44* for cloning of pFA20
pGAD_03223_fw	atggagtacccatacgacgtaccagattacgctcatATGGAAGCGGGCGATTCTGC	amplification of *pro44* CDS for cloning of yeast two-hybrid vector
pGAD_03223fl_rv	cccgtatcgatgcccacccgggtggaattcCTAGTGTCGATCGTCGGTGG	amplification of *pro44* CDS for cloning of yeast two-hybrid vector
pGBK_03223_fw	gaggagcagaagctgatctcagaggaggacctgcatATGGAAGCGGGCGATTCTGC	amplification of *pro44* CDS for cloning of yeast two-hybrid vector
pGBK_03223fl_rv	tgcggccgctgcaggtcgacggatccccgggaattcCTAGTGTCGATCGTCGGTGG	amplification of *pro44* CDS for cloning of yeast two-hybrid vector
SMU8399-for	TCTTCAGCCTCTGCCTTGGT	RT-qPCR *pks4*
SMU8399-rev	GCGATTCGATCCTCATCTCC	RT-qPCR *pks4*
SSU1	ATCCAAGGAAGGCAGCAGGC	RT-qPCR SSU-rRNA
SSU2	TGGAGCTGGATTTACCGCG	RT-qPCR SSU-rRNA
app-for2	GGAGATAGCTGGAGGGCTGA	RT-qPCR *app*

app-rev2	ATCTCGGGCTGACTTCCATC	RT-qPCR *app*
SMAC_09533_for2	CGCGTGCATCAGTCCGAAAT	RT-qPCR *SMAC_09533*
SMAC_09533_rev2	CCTAGACACTTTCCCGCGGT	RT-qPCR *SMAC_09533*
SMAC_09345_for	ACCGACAAGAACCTGCCCTC	RT-qPCR *SMAC_09345*
SMAC_09345_rev	CGTCTCACCAGCAACCCAAG	RT-qPCR *SMAC_09345*

[a]Culture collection: Lehrstuhl für Allgemeine und Molekulare Botanik, Ruhr-Universität, Bochum, Germany
