# Supplementary material for: The transcription factor PRO44 and the histone chaperone ASF1 regulate distinct aspects of multicellular development in the filamentous fungus Sordaria macrospora
Source: BMC Genet. 2018 Dec 13;19:112. doi: 10.1186/s12863-018-0702-z (PMC6293562; doi:10.1186/s12863-018-0702-z)
Supplement: Supplementary file 20 — Table S5. Plasmids used in this study. (PDF 270 kb) [file 12863_2018_702_MOESM20_ESM.pdf]

Table S5. Plasmids used in this study**.**

Plasmid	Characteristics	Reference or source
pDS23	PtrpC::*nat*, Pgpd::*egfp*::TtrpC	Schindler and Nowrousian (2014)
pBH2B	PtrpC::*hph*, Pgpd::*hh2b*::*mKalama1*::TtrpC	I. Teichert, unpublished data, based on pmKalama1 (Kück et al. 2009)
pRH2B	PtrpC::*hph*, Pgpd::*hh2b*::*tdTomato*::TtrpC	Teichert et al. (2014)
pKO-3223	1 kb upstream and 1 kb downstream regions of *S. macrospora pro44* flanking the PtrpC::*hph* cassette	this study
pKO_SMAC_02795	1 kb upstream and 1 kb downstream regions of *S. macrospora cdp1* flanking the PtrpC::*hph* cassette	this study
pKO_SMAC_09436	1 kb upstream and 1 kb downstream regions of *S. macrospora asm2*flanking the PtrpC::*hph* cassette	this study
pFA20	PtrpC::*nat*, Ppro44::*egfp*::*pro44*::Tpro44	this study
pFA30	PtrpC::*nat*, Pgpd::*ntap*::*pro44*::TtrpC	this study
pCAC2-EGFP	PtrpC::*nat*, Pgpd::*cac2*::*egfp*::TtrpC	this study
pRTT106-EGFP	PtrpC::*nat*, Pgpd::*rtt106*::*egfp*::TtrpC	this study
pSMAC_02795_EGFP	PtrpC::*nat*, Pgpd::*cdp1*::*egfp*::TtrpC	this study
pN_GFP-9436	PtrpC::*nat*, Pasm2::*egfp*::*asm2*::Tasm2	this study
pRSnat-pro44-NA	PtrpC::*nat*, Ppro44::*pro44*::Tpro44	this study
pGADT7	*ampR*, *leu2*, Padh1::*gal4AD*	Clontech/Takara Bio
pGBKT7	*kanR*, *trp1*, Padh1::*gal4BD*	Clontech/Takara Bio
pGAD_03223-A	*pro44* cDNA A in pGADT7	this study
pGAD_03223-B	*pro44* cDNA B in pGADT7	this study
pGAD_03223-C	*pro44* cDNA C in pGADT7	this study
pGBK_03223-A	*pro44* cDNA A in pGBKT7	this study
pGBK_03223-B	*pro44* cDNA B in pGBKT7	this study
pGBK_03223-C	*pro44* cDNA C in pGBKT7	this study

References:

Kück U, Pöggeler S, Nowrousian M, Nolting N, Engh I (2009) *Sordaria macrospora*, a model system for fungal development. In: Anke T, Weber D (eds.) The Mycota XV, Physiology and Genetics. Springer, Berlin, Heidelberg pp. 17-39

Schindler D, Nowrousian M (2014) The polyketide synthase gene *pks4* is essential for sexual development and regulates fruiting body morphology in *Sordaria macrospora*. Fungal Genet Biol 68:48-59

Teichert I, Steffens EK, Schnaß N, Fränzel B, Krisp C, Wolters DA, Kück U (2014) PRO40 is a scaffold protein of the cell wall integrity pathway, linking the MAP kinase module to the upstream activator protein kinase C. PLoS Genet 10:e1004582
